# Programmable biomolecular switches for rewiring flux in *Escherichia coli*

**DOI:** 10.1038/s41467-019-11793-7

**Published:** 2019-08-21

**Authors:** Cong Gao, Jianshen Hou, Peng Xu, Liang Guo, Xiulai Chen, Guipeng Hu, Chao Ye, Harley Edwards, Jian Chen, Wei Chen, Liming Liu

**Affiliations:** 1 State Key Laboratory of Food Science and Technology, Jiangnan University, Wuxi, 214122 China; 2 Key Laboratory of Industrial Biotechnology, Ministry of Education, Jiangnan University, Wuxi, 214122 China; 3 Chemical, Biochemical and Environmental Engineering, University of Maryland Baltimore County, Baltimore, Maryland 21250 USA; 4 National Engineering Laboratory for Cereal Fermentation Technology, Jiangnan University, Wuxi, 214122 China

**Keywords:** Metabolic engineering, Applied microbiology, Genetic circuit engineering, Synthetic biology

## Abstract

Synthetic biology aims to develop programmable tools to perform complex functions such as redistributing metabolic flux in industrial microorganisms. However, development of protein-level circuits is limited by availability of designable, orthogonal, and composable tools. Here, with the aid of engineered viral proteases and proteolytic signals, we build two sets of controllable protein units, which can be rationally configured to three tools. Using a protease-based dynamic regulation circuit to fine-tune metabolic flow, we achieve 12.63 g L^−1^ shikimate titer in minimal medium without inducer. In addition, the carbon catabolite repression is alleviated by protease-based inverter-mediated flux redistribution under multiple carbon sources. By coordinating reaction rate using a protease-based oscillator in *E. coli*, we achieve d-xylonate productivity of 7.12 g L^−1^ h^−1^ with a titer of 199.44 g L^−1^. These results highlight the applicability of programmable protein switches to metabolic engineering for valuable chemicals production.

## Abstract (web summary)

Current flux rewiring technologies in metabolic engineering are mainly transcriptional regulation. Here, the authors build two sets of controllable protein units using engineered viral proteases and proteolytic signals, and utilize for increasing titers of shikimate and D-xylonate in *E. coli*.

## Introduction

Engineering microbial cell factory to produce valuable chemicals from renewable feedstocks plays an essential role to implement sustainability^1,2^. To maximize product titer, yield, and productivity, metabolic flux needs to be finely reprogrammed without disrupting cellular homeostasis^3^. Current flux rewiring technologies have centered on the transcriptional-level regulation because of the ease and success of sophisticated engineering of metabolite-responsive transcriptional factors^4,5^. Despite unprecedented achievements making by transcriptional regulation^6,7^, the associated long response time can lead to faulted genetic circuits and ultimately fail to control metabolic flux^8^. Recently, multilayer regulation involving both transcriptional- and protein-level reprogramming is reported^9–11^. Protein-level regulation in those systems relies on the modification of target proteins with degradation tags or degrons^12–14^. However, these systems suffer from a number of issues, including tunability and orthogonality, and the lack of protein–protein interaction regulators have limited the ability to design and engineer fast-response biomolecular switches that can rapidly reprogram metabolic flux and persistently maintain cellular homeostasis.

In this study, viral proteases that specifically recognize and cleave short cognate target sites are applied in combination with proteolytic signals to control protein stability and residence time in *Escherichia coli*. To unravel the design principles underlying protease-based metabolic switches, we have developed a synthetic biology toolbox, including protease-based dynamic regulation circuit (pbDRC), protease-based inverter (pbI), and protease-based oscillator (pbO), to achieve fast-response and tunable control of metabolic flux. These rationally designed switches exhibit superior applicability for the regulation of metabolic flux and improvement of metabolite production in the industrial workhorse *E. coli*.

## Results

### Protease-based protein regulatory unit construction

**Fig. 1:**
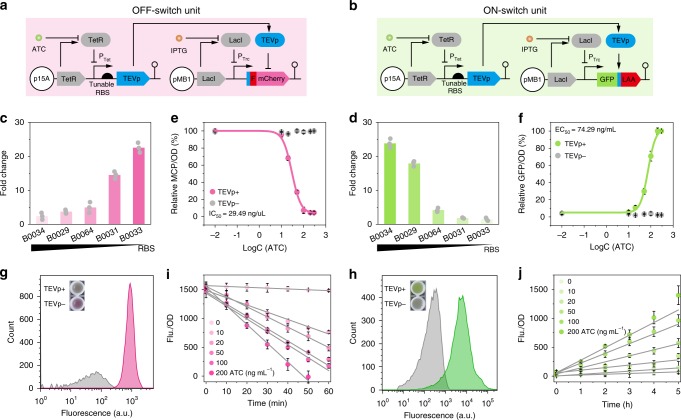
Basic protein regulatory unit design and characterization. **a**, **b** Design of the OFF-switch (**a**) and ON-switch (**b**) regulatory unit. **c**, **d** The dynamic range of reporter protein abundance by varying TEVp expression with ribosome binding site (RBS) variants in OFF-switch unit (**c**) and ON-switch unit (**d**). **e** Dose–response curve of OFF-switch unit (with B0033 RBS controlled TEVp) by varying inducer anhydrotetracycline (ATC) concentration. Nonlinear DoseResp model was utilized to fit the data shown by the magenta lines. IC_50_, half maximal inhibitory concentration. **f** Dose–response curve of ON-switch unit (with B0034 RBS controlled TEVp). Nonlinear DoseResp model was utilized to fit the data shown by the green lines. EC_50_, effective inducer concentration. **g** Flow cytometry diagrams of the control and the diagrams of the OFF-switch unit are represented by red tone traces and gray tone traces, respectively. **h** Flow cytometry diagrams of the control and the diagrams of the ON-switch unit are represented by gray tone traces and green tone traces, respectively. **i** The kinetic of OFF-switch unit (with B0033 RBS controlled TEVp) by varying ATC concentration in Luria-Bertani (LB) medium. **j** The kinetic of ON-switch unit (with B0034 RBS controlled TEVp) by varying ATC concentration in LB medium. TEVp+/TEVp− represents samples with (+) or without (−) ATC. Values are shown as mean ± SD from three biological replicates. Source data of Fig. 1c–f, 1i, and 1j are provided in Source Data file

To construct bifunctional switches that enable *E. coli* to accumulate or degrade target proteins when reprogramming metabolic flux, two basic protein regulatory units (including OFF-switch unit and ON-switch unit) were constructed. The OFF-switch unit consists of a protease that exposes the N-degron on the modified mCherry and drives its degradation (Fig. 1a). The ON-switch unit contains a protease that specifically removes the degradation tag of modified green fluorescent protein (GFP) and protects it from degradation (Fig. 1b). Depending on the configuration and orientation of the proteolytic tag and the protease cleavage tag, this protease-based switch demonstrated selective ON–OFF control of protein degradation.

To optimize the dynamic range of these units, we constructed a library of tobacco etch virus protease (TEVp) expression variants with different strength of RBSs (Supplementary Fig. 1). The proteolytic activity was screened, in which a 22.5-fold mCherry protein decrease in OFF-switch unit (Fig. 1c) and a 23.8-fold GFP increase in ON-switch unit was achieved (Fig. 1d). Kinetic analysis indicated that the processes of protein degradation and accumulation could be increased with higher inducer concentration, demonstrating that these basic protein regulatory units were tunable (Fig. 1e, f). Flow cytometry results demonstrated that both the protease-based ON/OFF-switch units exhibited good population homogeneity (Fig. 1g, h). To test system specificity, non-targeted mCherry protein was fused in both regulatory units. The expression of TEVp was able to downregulate the abundance of mVenus protein (yellow fluorescent protein (YFP)) by 27.2-fold in the OFF-switch unit and upregulated GFP abundance by 50.7-fold, which indicated that the ON/OFF-switch had excellent orthogonality in multi-protein processing (Supplementary Fig. 2). In vivo kinetic results showed that the half-life of protein degradation in the OFF-switch unit ranged from 25 to 60 min, with a dead time of <10 min (Fig. 1i, j). Moreover, compared with the commonly used LacI-IPTG-inducible expression system, they also demonstrated perfect dose–response curve (Supplementary Fig. 3). Those results indicated that the basic protein regulatory units were highly specific and quick in response.

### pbDRC design and characterization

**Fig. 2:**
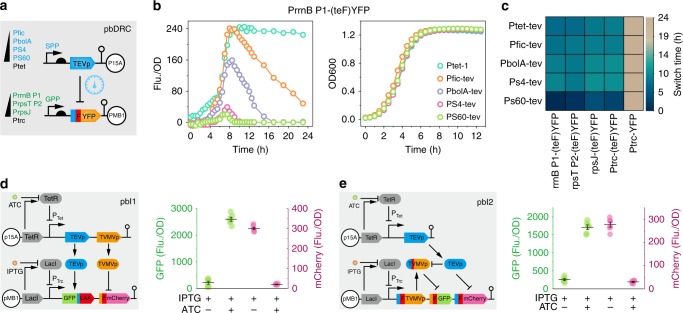
Design and characterization of pbDRC and pbI. **a** The pbDRC design. The time difference in the initiation of gene transcription by different physiological-dependent promoters provide prescribed switch time in controlling protein abundance. **b** The fluorescence abundance curve of strains with promoter rrnB P1-driven degradable YFP and SPP-driven TEVp. Ptet-1 represents a control plasmid without expression of TEVp. Cell growth curve of different pbDRC combinations (right). **c** Switch time in different promoter combination strains. Ptet-tev represents constitutively expressed TEVp. Ptrc-(teF)YFP and Ptrc-YFP represents constitutively expressed YFP with or without degradation signal. **d** First approach for pbI design. Two orthogonal proteases were co-expressed on a trigger plasmid. In the report plasmid, a TEVp recognition site was inserted between GFP and degradation tag LAA, and a TVMVp recognition site was inserted ahead of mCherry and a phenylalanine degron (F degradation signal). After expression of proteases, the degradable GFP became stable, whereas the stable mCherry became unstable. **e** Second approach for pbI design. TEVp was expressed on a trigger plasmid; TEVp-repressed TVMVp, TVMVp-repressed GFP, and TEVp-repressed mCherry were co-expressed on a report plasmid. After the TEVp expression, the degradable GFP became stable, whereas the stable mCherry became unstable. The dark line indicates the mean values from six parallel samples. Error bars mean ± SE. Source data of Fig. 2b–e are provided in Source Data file

Based on OFF-switch unit, the first tool we constructed was pbDRC (Fig. 2a). Both stationary phase promoter (SPP) and growth phase promoter (GPP) were contained in this circuit. Gene expression under the control of SPP will be repressed before the cell enters into stationary phase, whereas gene expression under the GPP will be repressed after the cell enters the stationary phase (Supplementary Fig. 4). To achieve tight control, a series of SPPs and GPPs were screened, among which four SPPs of different strengths (fic, bolA, S4, and S60) and three GPPs of different strengths (rrnB P1, rpsT P2, rpsJ) were selected (Supplementary Fig. 5).

Upon co-expression of SPP-driven TEVp and GPP-driven YFP, the resulting strains exhibited a variety of turning point in the reporter protein ranging from 7 to 10 h (Fig. 2b, c). Higher strength SPPs resulted in shorter switch time and lower reporter accumulation (Supplementary Fig. 6). To investigate the role of protease in pbDRC, constitutively expressed TEVp by promotor Ptet was co-expressed with either wild-type YFP or degradable YFP. Without degradation signal, the accumulation of wild-type YFP lasted for more than 24 h. However, a much shorter switch time (<4 h) in strains with degradable YFP demonstrated that TEVp-mediated proteolysis is indeed functional in the pbDRC (Fig. 2c). Furthermore, the growth curve confirmed that pbDRC had negligible pressure on cell growth (Fig. 2b). Thus, via a simple modification of promoters, YFP abundance could be dynamically controlled at different switch times by the pbDRC tool.

### pbI design and characterization

Based on ON/OFF-switch unit, we attempted to construct a second biomolecular tool pbI. First, we tested the direct introduction of one protease to control the different fates of GFP and mCherry. Although the introduction of TEVp could tune the abundance of mCherry and GFP, we observed that a higher level of TEVp was beneficial for tuning GFP accumulation but was unable to effectively modulate mCherry degradation (Supplementary Fig. 7). This result suggested that there was a trade-off between protein accumulation and degradation, which could not be addressed by simply introducing one protease. Thus, to facilitate unit compatibility, the pbI tool should contain proteases with equivalent functions but different specificities. Our designed pbI consisted of two orthogonal proteases, TEVp and TVMVp (tobacco vein mottling virus protease), to tightly control the abundance of the reporters (GFP and mCherry) (Supplementary Fig. 8).

Two types of pbIs were constructed (Fig. 2d, e). The first pbI consisted of two orthogonal proteases to control reporter level in OFF-switch unit or ON-switch unit (Fig. 2d). The second pbI contained an additional layered OFF-switch unit connected with either ON-switch unit or OFF-switch unit (Fig. 2e and Supplementary Fig. 9). Inversion of GFP and mCherry abundance was observed in both types of pbIs, demonstrating pbIs exhibited good composability in protein circuit construction.

### pbO design and characterization

**Fig. 3:**
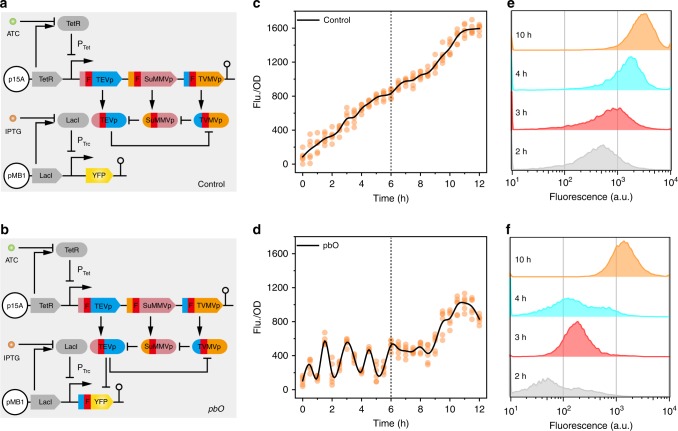
The pbO design and characterization. **a** Control system without YFP degradation. **b** Three proteases trigger oscillator system. **c**, **d** Characterization of fluorescence curve of control strain (**c**) and pbO strain (**d**). Black trace was the mean value of discrete data points measured from six separate colonies. **e**, **f** Flow cytometry measurements of control strain (**e**) and pbO strain (**f**) at different time points. Source data of Fig. 3c and 3d are provided in Source Data file

We also constructed a third biomolecular tool pbO. Inspired by the classical example of the repressilator^15^ (Supplementary Fig. 10), we hypothesized that the reporter protein abundance could be periodically modulated with periodical protease input. Our oscillator ring structure consisted of three orthogonal—TEVp, TVMVp, and SuMMVp (sunflower mild mosaic virus protease)^10^ (Supplementary Fig. 10). Moreover, each was modified by fusing their N terminus with a degron and other protease cleavage sites that could be specifically recognized and degraded by corresponding proteases. Reporter expression results indicated that modified proteases also exhibited excellent orthogonality and specificity (Supplementary Fig. 11). To further validate this hypothesis, either an individually expressed TEVp (Supplementary Fig. 12) or all three proteases co-expressed at the same strength (Fig. 3b) were incorporated into the pbO as a trigger. To analyze the effect of proteases cascade degradation on fluorescent protein accumulation, a control strain in which YFP without any protease cleavage sites co-expressed with all three proteases was also constructed (Fig. 3a).

**Fig. 4:**
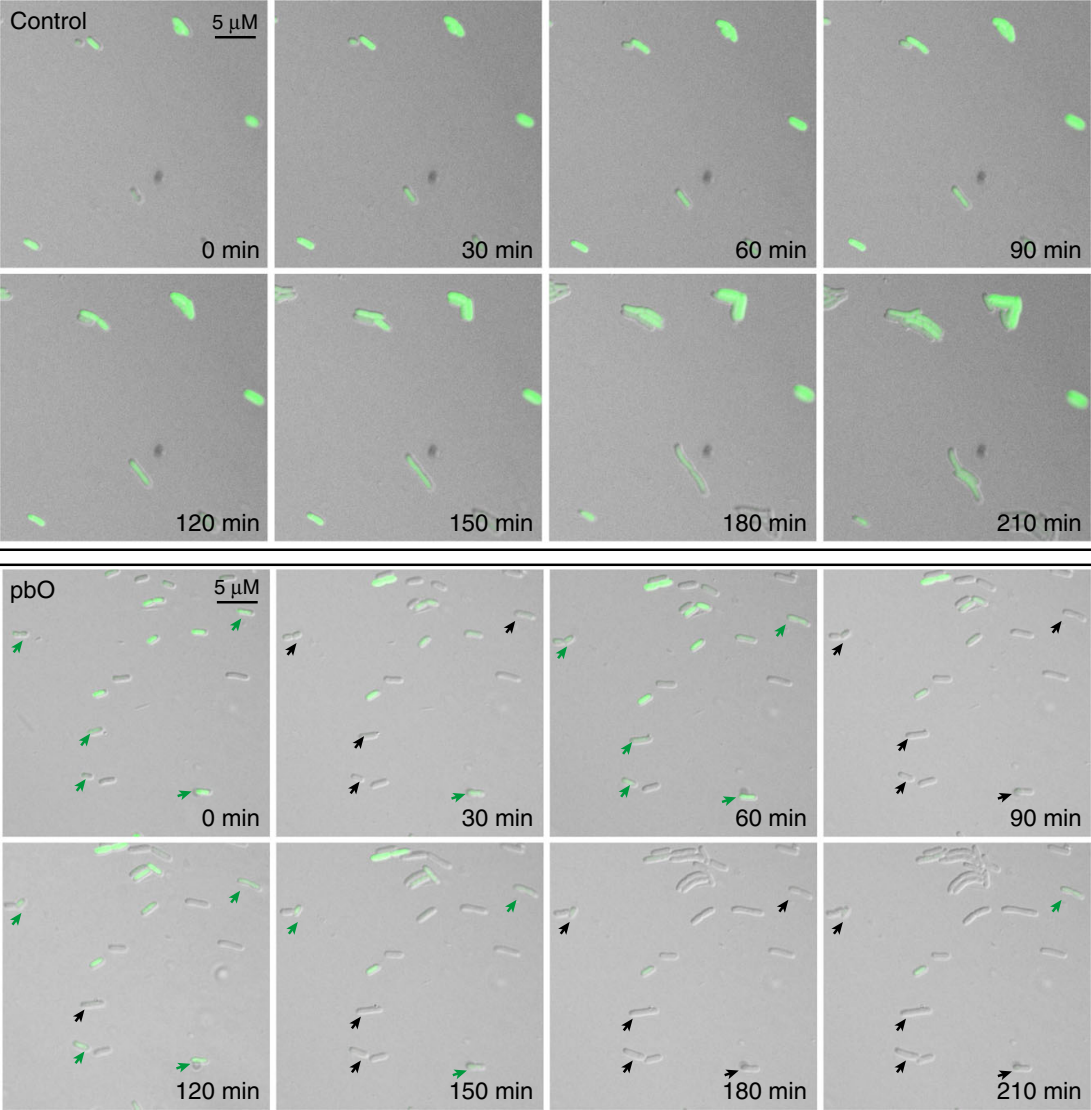
The single-cell fluorescence microscopy of the control and pbO system. Engineered cells were immobilized on LB agar on a microscope slide, and imaged every 30 min using microscope. Green arrows indicate the formation of fluorescence, whereas black arrows indicate the loss of fluorescence. Compared with the control strains, the arrow-marked pbO cells can exhibit periodic fluorescence loss and revival as time progresses. The reason for the loss of fluorescence after 150 min could be explained by oxygen deficit. Under the anaerobic microenvironment, the newly synthesized fluorescence protein is difficult to be oxidized to mature. Source data are provided in Source Data file

Then, we profiled the YFP abundance by continuously detecting the fluorescence intensity in each case. Compared with the control, in the case with different expression level of proteases, i.e., TEVp was expressed on a low copy number plasmid, whereas other two proteases co-expressed on a high copy number plasmid, reporter gene expression only exhibited two indistinctive peaks in the first 2.5 h before a dramatic increase (Supplementary Fig. 12). However, with all three proteases acting together as the trigger, i.e., the modified TEVp, TVMVp, and SuMMVp were co-expressed on a low copy number plasmid, periodical YFP peaks could be observed in the first 6 h culture (Fig. 3c, d). A loss of oscillations and synchronization was detected after 6 h possibly due to the stochasticity of gene expression and population heterogeneity in shake flask. This hypothesis was further verified by flow cytometry results, in which population heterogeneity could be found during the oscillation period (the first 4 h culture). Nevertheless, we noticed that the YFP expression level is fluctuant, with nearly half strength of that from the control strain after 12 h culture. Moreover, single-cell fluorescence microscopy results indicated that individual pbO cells could exhibit sustained oscillations compared with the control strains (Fig. 4). Collectively, those results indicated that pbO can be constructed by periodical protease input and fine-tuning is needed in the future to achieve a perfect oscillatory expression.

### Dynamic flux regulation in shikimate production using pbDRC

**Fig. 5:**
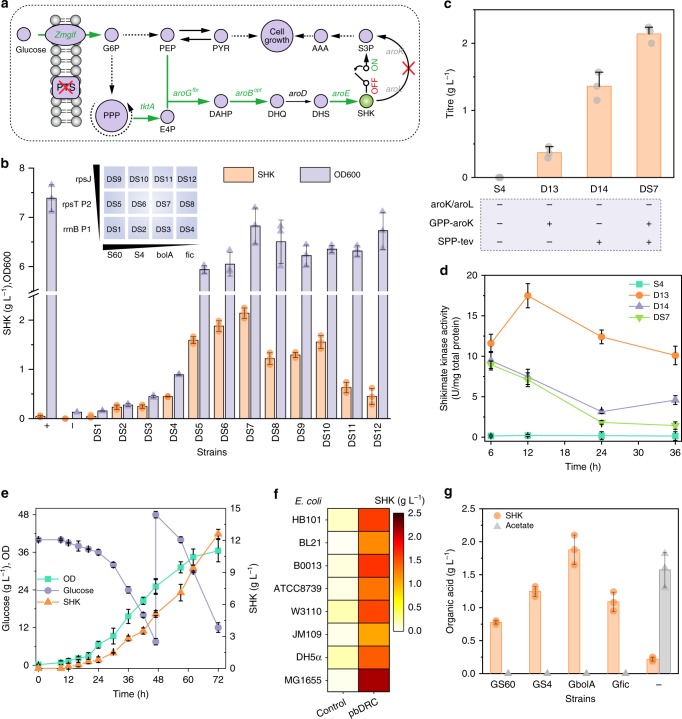
Dynamic regulation for shikimate production. **a** Engineering shikimate biosynthetic pathway in chassis *E. coli* strain S4. Red cross indicated pathway blocking. Genes that are bold were overexpressed. **b** Effect of different combinations of the pbDRC on titer and cell growth (72 h). + and − represent chassis S4 cultured in NBS medium with (+) or without (−) AAA supplement. Insert was orthogonality matrix for strains construction. **c** Titer comparison of different strains in NBS medium without AAA supplement. Insert: aroK/aroL represents the genomic expression of shikimate kinase I/II; GPP-aroK represents the expression of shikimate kinase I under the control of GPP; SPP-tev represents a SPP-based expression of TEVp with a constitutive expression of shikimate kinase I. **d** Evaluation of shikimate kinase I degradation in different strains during shikimate production. **e** Shikimate production of engineered strain DS7 in bioreactor. **f** Titer comparison of different strains and corresponding engineered strains with pbDRC system (72 h). **g** Genomic-scale dynamic pathway modulation of shikimate production (72 h). – represents strain without expressing TEVp. Values are shown as mean ± SD (*n* = 3). aroB^opt^, DHQ synthase with first eight codons optimized; aroD, DHQ dehydratase; aroE, shikimate dehydrogenase; aroG^fbr^, feedback-resistant mutants of DAHP synthase (D146N); aroK/aroL, shikimate kinase I/II; DAHP, 3-deoxy-D-arabino-heptulosonate-7-phosphate; DHQ, 3-dehydroquinic acid; DHS, 3-dehydroshikimate; E4P, erythrose-4-phosphate; G3P, glyceraldehyde 3-phosphate; G6P, glucose 6-phosphate; PEP, phosphoenolpyruvate; PTS, phosphotransferase system; PP pathway, pentose phosphate pathway; PYR, pyruvate; SHK, shikimate; S3P, shikimate-3-phosphate; tktA, transketolase A; Zmglf, glucose facilitator from *Z. mobilis*. Source data of Fig. 5b–g are provided in Source Data file

We started off by applying the pbDRC to control carbon flow. Shikimate (SHK), an important starting material in treating influenza^16^, is traditionally produced by blocking metabolic flow from SHK to SHK-3-phosphate (S3P) (Fig. 5a). This eventually leads to the interruption of endogenous aromatic amino acid (AAA) synthesis and the sacrifice of cell growth. Exogenous AAAs can be supplemented to avoid defects in cell growth, which inevitably increases the manufacturing costs of SHK^16^. An alternative solution is to decouple cell growth from SHK production^11^. We therefore used an engineered *E. coli* S4 as a chassis, in which SHK kinases were deleted, phosphotransferase system was replaced by glucose facilitator from *Zymomonas mobilis*, and pathway enzymes encoding by *tktA*, *aroG*, *aroB*, and *aroE* were overexpressed (Fig. 5a and Table 1). This strain exhibited growth defects in New Brunswick Scientific (NBS) minimal medium. Next, we engineered SPP-controlled TEVp into *E. coli* S4 to regulate GPP-controlled *aroK* (SHK kinase I), which converts SHK to S3P in the metabolic pathway. Strains (DS1–DS12) containing different combinations of SPPs and GPPs were assayed in NBS minimal medium without the addition of AAA and inducers, among which strain DS7 with a combination of promotor rpsT P2 and bolA exhibited a SHK titer increase from undetectable to 2.14 g L^−1^ without cell growth sacrifice (Fig. 5b and Supplementary Figs. 13, 14).

**Table 1:** Bacterial strains used for tool applications

Strains	Description	Source
S3	*E. coli* MG1655, Δ*ptsHIcrr*::*Zmglf*, Δ*aroL*::*tktA*	Hu et al.^42^
S4	S3, Δ*aroK*	This study
DS1/2/3/4	S4: pGABE-BK, pS60-*tev*/pS4-*tev*/pbolA-*tev*/pfic-*tev*	This study
DS5/6/7/8	S4: pGABE-TK, pS60-*tev*/pS4-*tev*/pbolA-*tev*/pfic-*tev*	This study
DS9/10/11/12	S4: pGABE-JK, pS60-*tev*/pS4-*tev*/pbolA-*tev*/pfic-*tev*	This study
DS13	S4: pGABE-TK, pTet-1	This study
DS14	S4: pGABEK, pbolA-*tev*	This study
GS60	S3, Δ*aroK*::prpsT P2-*(teF)aroK*, pS60-*tev*	This study
GS4	S3, Δ*aroK*::prpsT P2-*(teF)aroK*, pS4-*tev*	This study
GbolA	S3, Δ*aroK*::prpsT P2-*(teF)aroK*, pbolA-*tev*	This study
Gfic	S3, Δ*aroK*::prpsT P2-*(teF)aroK*, pfic-*tev*	This study
X1	*E. coli* BL21(DE3), ∆*XylAB*	This study
X2	X1, ∆*ptsI*	This study
X3	X2, pTrcHisA-*(teF)ptsI*	This study
X4	X2, pTet-33*tev*, pTrcHisA*-(tvF)ccxylB-(teF)ptsI-(teF)tvmv*	This study
XP	X1, pTet-1, pTrcHisA-*ccxylB*	This study
XN	X1, pTet-1, pTrcHisA-*ccxylB-(teF)ccxylC*	This study
XO	X1, pTet-*(tvF)summv-(suF)tev-(teF)tvmv*, pTrcHisA-*ccxylB-(teF)ccxylC*	This study

To show the relationship between target enzyme degradation and titer improvement, the SHK kinase activity of four different strains including S4, D13 (SHK kinase I was expressed under the control of GPP, P_rpsT P2_), D14 (SHK kinase I was constitutively expressed, but would be degraded by SPP-based induced TEVp), and DS7 (GPP-based repression of SHK kinase I combined with SPP-based induced degradation) were evaluated during the SHK production (Supplementary Fig. 14 and Table 1). As shown in Fig. 5d, SHK kinase could be degraded in the same manner as the fluorescent protein, given the fact that the SHK kinase activity of strain DS7 at 36 h was only 0.16-fold of that at 6 h. Moreover, we observed a direct negative correlation between SHK kinase activity and SHK titer (Fig. 5c).

To determine the applicability of pbDRC in bench-top bioreactors, the performance of strain DS7 in a 5 L fermenter was investigated. Compared with that of shaker flasks, a 5.9-fold higher SHK titer (12.63 g L^−1^, yield of 0.19 g g^−1^ glucose) was achieved (Fig. 5e). Moreover, to evaluate pbDRC as a universal tool, other seven *E. coli* variants including DH5α, JM109, W3110, ATCC 8739, B0013 (derived from *E. coli* K-12); BL21 (derived from *E. coli* B); and HB101 (a hybrid of the *E. coli* K-12 and B) were engineered at the same sites, according to strain DS7. As shown in Fig. 5f, all the pbDRC-containing strains achieved increased SHK titer ranging from 7.3- to 42.8-fold compared with that of the control strains, respectively. Furthermore, genomic integration of pbDRC was also attempted in *E. coli* MG1655 by replacing the wild-type *aroK* with the rpsT P2 promoter-driven cleavable *aroK*. All strains with SPP-controlled TEVp exhibited a 3.6- to 8.5-fold titer (0.79–1.88 g L^−1^) without acetate accumulation in comparison with the control strain (Fig. 5g). Overall, pbDRC provided dynamic, robust, strain-independent regulation of metabolic flux flow at scales of industrial relevance.

### Flux direction regulation in d-xylonate production using pbI

The second application was to control the direction of carbon flux using pbI. d-xylonate, one of the top 30 high-value chemicals, was currently produced with pure d-xylose, which greatly increased production costs^17^. Attempts at decreasing the production cost have focused on using low-cost substrates, such as cellulose hydrolysate^18^. However, the major obstacle in this process was carbon catabolite repression, which prolongs the uptake of d-xylose when enough glucose is present^19^ (Supplementary Fig. 15).

**Fig. 6:**
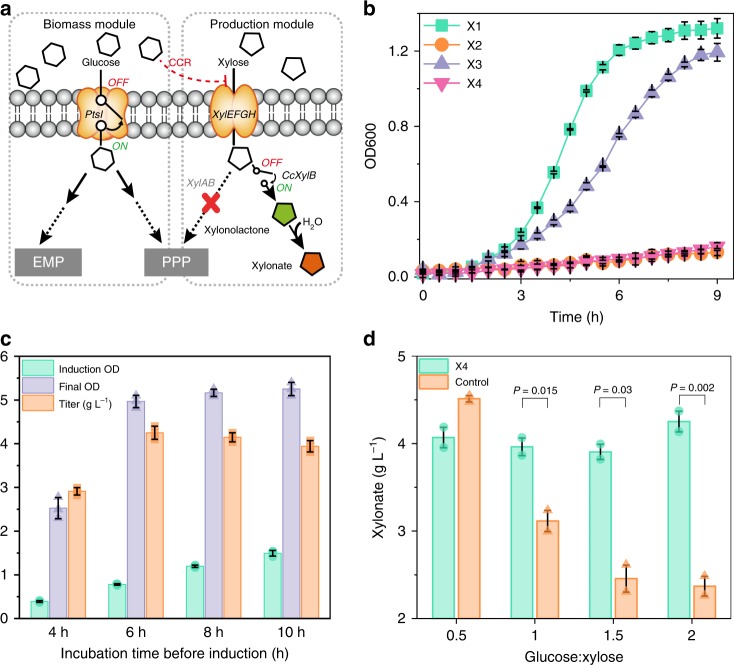
Application of pbI in d-xylonate production. **a** d-Xylonate metabolic pathways in *E. coli* with pbI. Red cross denotes gene disruption. **b** Growth curve of different strains in NBS minimal medium with glucose as carbon source (0.05 mM IPTG was pre-added to the medium of strain X3; 0.05 mM IPTG and 100 ng mL^−1^ ATC was pre-added to the medium of strain X4). Data point indicates the mean values from three parallel samples in 96-microplate at 37 °C. **c** Induction time evaluation of strain X4. Values are shown as mean ± SD (*n* = 3). **d** Titer comparison of control strain (wild-type strain with d-xylose dehydrogenase overexpression) and X4 strain. Anhydrotetracycline (200 ng mL^−1^) was added at exponential growth period (6 h). Values are shown as mean ± SD (*n* = 2). Significance (*p*-value) was evaluated by two-sided *t*-test. CCR, carbon catabolite repression; CcxylB, *Caulobacter crescentus* d-xylose dehydrogenase; EMP, embden-meyerhof-parnas pathway; PPP, pentose phosphate pathway; PtsI, phosphotransferase enzyme I; XylA, xylose isomerase; XylB, xylulose kinase; XylEFGH, xylose transport proteins. Source data of Fig. 6b–d are provided in Source Data file

A promising strategy to address this issue is to block glucose utilization after the biomass reaches a certain density using pbI. To test this, a chassis of *E. coli* X3 was constructed by introducing cleavable phosphotransferase enzyme I (PTSI) in a d-xylose, d-xylonate, and glucose catabolic defects strain X2 (Fig. 6a and Table 1). Then, a pbI, which contained TVMVp-cleavable d-xylose dehydrogenase, TEVp-cleavable TVMVp, TEVp-cleavable PTSI, and TEVp, were co-expressed in strain X3 (Table 1 and Supplementary Fig. 14). The resulted strain X4 exhibited a growth defect phenotype in the ATC pre-added glucose-NBS minimal medium. This phenotype was consistent with a PTS defect strain X2, suggesting that the activity of phosphotransferase system can be artificially controlled by introducing pbI (Fig. 6b). Subsequently, we tested the effect of different induction time on strain growth and xylonate production. Result of the induction time in strain X4 demonstrated that higher d-xylonate production (4.25 g L^−1^) could be achieved when induced after 6 h of incubation (OD_600_ = 0.78) (Fig. 6c). On this basis, strain X4 exhibited comparable d-xylonate titer under different sugar ratios in mixed sugar culture conditions. Notably, when the sugar ratio was controlled at 2 (glucose vs. xylose), a 1.79-fold titer increase (4.25 g L^−1^) was achieved over that of the control strain (Fig. 6d). Those results revealed that pbI effectively rerouted the direction of metabolic flux, which has a great potential in d-xylonate or other high-value compounds production from mix substrates.

### Flux rate regulation in d-xylonate production using pbO

The third application is using pbO to tune carbon flux rate. Previous studies suggested that a higher d-xylonate titer could be achieved at an early stage of culture by co-expression of xylose dehydrogenase and lactonase^20^. However, given the fact that lactonase-mediated hydrolysis of d-xylonolactone to d-xylonate acidifies the cytoplasm, thereby decreasing cell viability, which ultimately compromises d-xylonate productivity^21^, the current d-xylonate titer and productivity produced by engineered *E. coli* was less commercially competitive by individual expression of xylose dehydrogenase (39.2 g L^−1^ with 1.09 g L^−1^ h^−1^)^22^ or simply by co-expressing xylose dehydrogenase and lactonase at a low level (108.2 g L^−1^ with 1.8 g L^−1^ h^−1^)^23^. Therefore, increasing d-xylonate titer relied on the precise control of the cascade reaction rate.

**Fig. 7:**
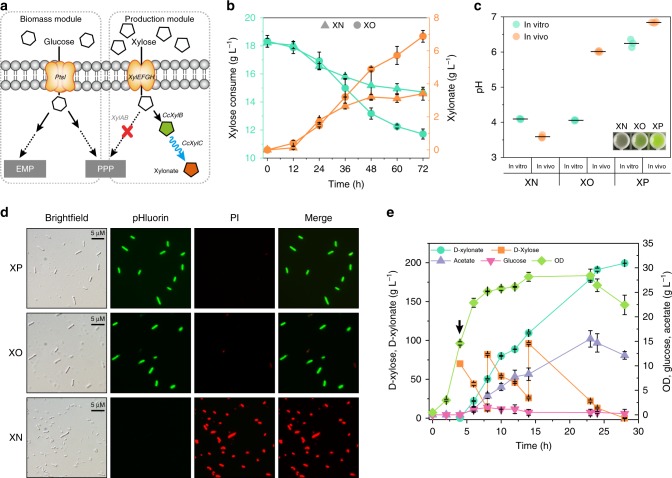
Application of pbO in d-xylonate production. **a** d-Xylonate metabolic pathways in *E. coli* with pbO. Red cross denotes gene disruption. Wavy line denotes metabolic flux was periodical slowed down. **b** Time course of strain XN (solid triangles) and XO (solid circles) in xylose consumption and xylonate production. **c** The comparison of in vivo and in vitro pH value between different strains. XO, strains with oscillator; XN, strains without oscillator; XP, strains with xylose dehydrogenase but without lactonase overexpression. Insert: photograph of samples from strain XN, XO, and XP. **d** A series of microscopy images of different strains in xylonate production (48 h). **e** d-xylonate production of strain XO in 5 L bioreactor. Black arrow indicates induction start time. Values are shown as mean ± SD (*n* = 3). CCR, carbon catabolite repression; CcxylC, *C. crescentus* d-xylono-lactone lactonase; PI, propidium iodide dye. Source data of Fig. 7b-e are provided in Source Data file

We reasoned that periodically slowing down the downstream reaction rate of the cascade should improve xylonate production without impairing cell viability (Fig. 7a). To validate this hypothesis, engineered strain X1 with d-xylonate and d-xylose catabolic defects was chosen as chassis for pbO demonstration (Table 1). By co-expressing xylose dehydrogenase, TEVp-cleavable lactonase, and trigger plasmid pTet-(tvF)*summv*-(suF)*tev*-(teF)*tvmv*, the resulting strain, XO, harboring pbO was constructed (Supplementary Fig. 14). As a result, a 2.02-fold titer (6.88 g L^−1^), with a yield of 1.04 g g^−1^ xylose, was achieved in the strain XO compared with that of the negative control strain XN (Table 1 and Supplementary Fig. 14), although they had similar biomass accumulation (Fig. 7b and Supplementary Fig. 16).

To investigate the mechanism underlying titer improvement, a pH reporter, pHluorin, from *Aequorea victoria* was expressed^21^. The *R*_410/470_ values obtained from cells containing pHluorin protein could be used to quantify the intracellular pH (in vivo pH), because they exhibited a biphasic relationship to pH from 3 to 8 (Supplementary Fig. 17). In addition to the strain XN, a positive control strain (XP) with xylose dehydrogenase but without lactonase overexpression was constructed (Supplementary Fig. 14). As shown in Fig. 7c, compared with XP, strains co-expressed with xylose dehydrogenase and lactonase (XN and XO) exhibited a good d-xylonate production capacity according to results from the extracellular pH (in vitro pH). Moreover, by introducing protein oscillator, intracellular pH (in vivo pH) homeostasis was achieved in strain XO, which further led to the observed increase in cell viability by more than two orders of magnitude in 36 h compared with that of the control strain XN (Fig. 7d and Supplementary Fig. 18). Lastly, culture conditions were optimized, in which a d-xylonate titer of 13.11 g L^−1^ was achieved using Terrific Broth (TB) medium at 37 °C in shaker flasks (Supplementary Fig. 19). Under such conditions, results of engineered XO strain in a 5 L fed-batch culture demonstrated the highest d-xylonate titer and productivity could reach 199.44 g L^−1^ and 7.12 g L^−1^ h^−1^, respectively (Fig. 7e). In summary, pbO provide an advantageous tool for precisely controlling metabolic events in an engineered cell factory.

## Discussion

In this study, we constructed two composable protein regulation units, which could be rationally applied in the design of three biomolecular toolkits—pbDRC, pbI, and pbO. Experimental results clearly demonstrated that these carbon flux regulation tools could perform sophisticated functions and exhibited good applicability in optimizing carbon flux under various scenarios of metabolic engineering practices.

The protease-based ON/OFF-switch exhibited good composability and extendibility in protein-level circuit construction. Compared with the transcriptional or posttranscriptional regulations reported in previous studies (the input relied on small-molecule inducers^24,25^ or nucleic acids^26,27^ and output depended on transcription factors or nucleic acid-interacting proteins), the input and output of protease-based circuits could be standardized to orthogonal proteases. Thus, by rationally configuring engineered viral proteases and proteolytic signals, distinct ON/OFF switches could be created and connected to each other to yield predictable behavior^28,29^. Recently, a series of protein circuits was constructed in mammalian cells by introducing leucine zipper motifs and split proteases^28,30^. Different from those studies, an alternative approach for constructing tunable protein circuits in *E. coli* was provided in this work by controlling the input protease dosage. Moreover, the types of protein circuits were further expanded by developing three protease-based biomolecular toolkits.

On the principle that enzyme activity is associated with its abundance, these tools could be used for precisely tuning carbon flux, direction, and rate. Base-pairing interactions at the transcriptional level could offer numerous programmable technologies, but there is a common drawback of prolonged response times^4,6,30^. Although proteases could respond rapidly to cleave and induce the degradation of target proteins^28,31^, its orthogonality with the host makes it difficult to sense metabolite concentration changes or the state of the host for making corresponding dynamic adjustments. In this study, we demonstrated that an intelligent circuit, pbDRC, could be constructed by additional integration of transcription-level regulation on protein-level circuit. By rational combinations of GPPs and SPPs to trigger the production of proteases to obtain ON or OFF status of metabolic proteins, we provided a valuable alternative to previous QS-dependent dynamic regulation system^11^. Moreover, due to the independence of homologous degradation machinery in *E. coli*, our pbDRC platform exhibited general applicability across *E. coli* strains and demonstrated robust performance at scales of industrial relevance.

Compared with previously reported transcriptional-level inverters that relied on CRISPRi^32,33^, zinc finger proteins, small RNAs^34^, and antisense RNA^35^, here, a pbI was constructed, which exhibited transcriptional-independent characteristics, i.e., protein functionality can be changed, even after the cognate mRNAs have been translated^36^. This could be beneficial for metabolic engineering cases where product synthesis competes with biomass formation of common precursors^37^. Furthermore, the rapid response kinetics involved in protein-level regulation enabled us to build inverters with broader dynamic ranges and precise time-switching functions than previously reported inverters^35^.

The inherent nonlinearity of protease input and protein degradation output can offer oscillatory regulation with shorter periods than transcriptional-level regulation. Previously, a classic ring oscillator was developed using three orthogonal transcriptional regulators with a cycle time of 160 ± 40 min^38^. In this study, with a shorter signal conduction chain, pbO achieved an oscillatory period closer to 90 min. Moreover, the robustness of our protein oscillator was improved by coordinating protease input, indicating that pbO display high levels of interconnectedness. In practical applications involving metabolic flux rate tuning, this protein oscillator exhibited unique application potential. Compared with the direct overexpression of lactonase, periodically regulating lactonase reaction rates using protein oscillators could achieve a 1.84-fold higher d-xylonate titer and 4-fold productivity greater than previous reports in engineered *E. coli*^23^. We expected that by tuning the input dose of protease, the frequency and intensity of oscillators could be further optimized to address metabolic engineering problems, such as the modulation of metabolic pathways involved in toxic intermediate accumulation^7^ or accurate synthesis of target chemicals with a specific molecular weight range^37^.

Although protease-based posttranslational regulation offers many advantages on rapid response, composability, and modularity^28,39^, some limitations should also be taken into account when constructing protein circuits. The first one is the ATP cost associated with proteolysis. As the unfolding and translocation of protein substrate are energetically unfavorable, the total ATP cost of ClpP-mediated proteolysis can be relatively high. For example, about 600 ATPs will be consumed in the degradation of a model substrate titin^I27^ domain^40^. This energy burden caused by introducing controllable proteolysis may affect both cell proliferation and genetic circuit output. Thus, it is necessary for decreasing intracellular resource occupancy by fine-tuning the output strength of artificial circuits in the design and implementation of protein circuits^39^. The second limitation is the queueing effect. When multiple proteins are designed to be degraded and competed for proteolytic resources, the ClpP machinery will be overloaded. As a result, the unexpected queueing coupling response can occur, which will decrease target protein degradation rate, affect the innate parameters of genetic parts and subsequently change the profile of circuits output^41^. One promising strategy to overcome queueing effect is to exploit heterologous proteolysis machinery, such as *Mesoplasma florum* Lon protease complexes^12^.

Transcriptional regulation systems are frequently used in metabolic engineering, most commonly for the overexpression of pathway enzymes and for tight regulation of toxic products. In this study, three protease-based circuit systems were able to complement current transcriptional regulation systems and expand their versatility by harnessing reprogrammable protein regulation units. With this protease-based synthetic biology toolbox, protease-based circuits involving protein-level regulation are expected to advance metabolic engineering and synthetic biology.

## Methods

### Strains and plasmids

Strains used for the application of protease-based tools in this study are listed in Table 1. All constructs used for tools development were generated using ligation cloning procedures from Takara Bio (Dalian, China) or one-step cloning kit procedure from Vazyme Biotech (Nanjing, China). A list of all genetic components and plasmids reported in this manuscript are included in Supplementary Data 1, Supplementary Fig. 20, and Supplementary Tables 2-7. DNA sequences of those plasmids can be found in Supplementary Data 1.

For SHK production, *E. coli* MG1655^42^ was used for chassis strain S4 construction. The gene *aroL* was genomically replaced by a *tktA* expression cassette under the control of promoter J23119 and RBS B0034. The PTS system (*ptsH*, *ptsI*, and *crr*, encoding HPr, EI, and EIIA^Glc^) was genomically replaced by a glucose facilitator protein gene, *Zmglf*, from *Z. mobilis*. *E. coli* strains B0013^43^, ATCC 8739^44^, W3110^45^, HB101, BL21, JM109, and DH5α were used for the universal verification of pbDRC with the same operations. To test the genomic level regulation of pbDRC, the N terminus of *aroK* was tagged with a TEVp site (cleavage site: ENLYFQ) followed by the F-degron (FLFVQ). This modified *aroK* was genomically inserted into the place of wild-type *aroK* under the control of promoter rpsT P2 in chassis S4. Pathway enzymes, including *aroG*^*fbr*^, *tktA*, *aroB*^*opt*^, and *aroE* were selected for constructing enzyme overexpression plasmid pGABE. The backbone plasmid was a constitutive expression plasmid pJ01 (GenBank accession MK234843) with a pMB1 replication origin. The gene *aroG*^*fbr*^, a feedback-resistant mutant involving D146N, was obtained by rapid PCR site-directed mutagenesis^46^. The gene *aroB*^*opt*^, a codon-optimized variant, was obtained by optimization of the first eight codons (ATG GAG CGT ATT GTC GTT ACT CTG)^47^. An improved solubility and autolysis inactivated TEVp mutant (T17S, L56V, N68D, I77V, S135G, S219V)^48^ was codon-optimized and expressed under the control of SPPs on a low copy number plasmid, pTet-1 (GenBank accession MK234848). The N terminus of *aroK* was tagged with a TEVp site followed by an F-degron. This modified *aroK* was inserted into the pathway enzyme overexpression plasmid pGABE under the control of GPPs, including P_rrnB P1_, P_rpsT P2_, and P_rpsJ_.

For d-xylonate production, chromosomal genes including *xylA* (encoding xylose isomerase), *xylB* (encoding xylulose kinase), and *ptsI* (encoding EI in PTS system) was knocked out in *E. coli* BL21(DE3). All of the above gene deletion or replacement operations were carried out using CRISPR/Cas9 technology^49^. The *xylB* gene from *C. crescentus* was codon optimized, chemically synthesized, and cloned into the pTrcHisA vector to obtain pTrcHisA-*ccxylB*. The *ptsI* gene was cloned from genomic PCR amplification products. On the basis of this, the N terminus of *xylB* was tagged with a TVMVp site (cleavage site: ETVRFQ) followed by a F-degron. The N terminus of *ptsI* and *tvmv* were tagged with a TEVp site followed by a F-degron. The above three gene segments were assembled into a pTrcHisA vector using a one-step cloning kit. The *xylC* gene from *C. crescentus* was codon optimized, chemically synthesized, and N terminus modified by tagging with a TEVp site followed by a F-degron. SuMMVp (cleavage site: EEIHLQ) was applied for constructing protein oscillators. Successful gene cloning was verified by colony PCR, restriction mapping, and direct nucleotide sequencing.

### Culture conditions

For SHK production in shaker flasks, seed cultures were grown overnight in LB medium at 37 °C and then transferred into 50 mL NBS minimal medium (3.5 g L^−1^ KH_2_PO_4_, 5.0 g L^−1^ K_2_HPO_4_, 3.5 g L^−1^ (NH_4_)_2_HPO_4_, 0.25 g L^−1^ MgSO_4_ ▪ 7H_2_O, 15 mg L^−1^ CaCl_2_ ▪ 2H_2_O, 0.5 mg L^−1^ vitamin B1, 1 mL L^−1^ trace element solution (1.6 g L^−1^ FeCl_3_, 0.2 g L^−1^ CoCl_2_ ▪ 6H_2_O, 0.1 g L^−1^ CuCl_2_, 0.2 g L^−1^ ZnCl_2_ ▪ 4H_2_O, 0.2 g L^−1^ NaMnO_4_, 0.05 g L^−1^ H_3_BO_3_, dissolve in 0.1 M HCl))^50^ containing 40 g L^−1^ glucose and supplemented with 100 mg mL^−1^ ampicillin and 30 mg mL^−1^ chloramphenicol. All cultures were grown in 250 mL shaker flasks at 200 r.p.m. and at 33 °C, which is the optimal temperature for SHK production.

For SHK production in bioreactors, *E. coli* strain DS7 harboring plasmids GABE-TK and pbolA-*tev* was first inoculated into 50 mL LB medium with 100 mg mL^−1^ ampicillin and 30 mg mL^−1^ chloramphenicol at 37 °C, with shaking at 200 r.p.m. for 12 h. Then, overnight seed cultures were collected, washed, and resuspended in NBS minimal medium containing 40 g L^−1^ glucose. The resulting seed cultures were transferred to a 5 L INFORS fermenter with NBS minimal medium at an initial OD_600_ of 0.05. The pH was maintained at 7 by automatically feeding 30% NH_4_OH and 2 M HCl. Air flow was set at 1 v.v.m. and the dissolved oxygen concentration was controlled above 30% saturation via cascade agitation (300–600 r.p.m.). Culture temperature was controlled at 33 °C. Glucose (40 g L^−1^) was added when the residual glucose fell below 10 g L^−1^. Samples were taken as required and analyzed by high-performance liquid chromatography (HPLC).

For d-xylonate production in shaker flasks, seed cultures were grown in 50 mL of LB medium in 250 mL shaker flasks at 37 °C on a rotary shaker at 200 r.p.m. overnight. Then, 5% (vol/vol) of seed culture was inoculated into 50 mL of NBS medium with 100 mg mL^−1^ ampicillin and 30 mg mL^−1^ chloramphenicol, and cultured at 37 °C, 200 r.p.m. When strain density reached 0.8, corresponding inducers (IPTG, ATC) were added. To optimize d-xylonate production, TB, 1.5 × LB and NBS medium were used and culture conducted at either 37 °C or 30 °C.

For d-xylonate production in bioreactors, seed cultures were prepared by incubating the strain XO in shaker flasks containing liquid LB medium overnight at 37 °C. Then, 5% (vol vol^−1^) of seed culture was inoculated into 2 L of the TB medium with 100 mg mL^−1^ ampicillin in a 5 L INFORS fermenter. The culture was first operated in a batch mode and the control settings were as follows: 37 °C, stirring speed 600 r.p.m., and airflow at 0.5 v.v.m. During the culture process, the pH was controlled at 7.0 via automated addition of 30% NH_4_OH and 2 M HCl. Antifoam 204 was added to prohibit foam development. When the dissolved oxygen started to increase at 4 h (OD_600_ = 14–15), 0.5 mM IPTG, 200 ng mL^−1^ anhydrotetracycline, and 70 g L^−1^ d-xylose were added for d-xylonate biosynthesis. Moreover, 500 g L^−1^ glucose was fed into the fermenter at a constant speed of 8 mL min^−1^ to achieve 2 g L^−1^ h^−1^ glucose supplementation until the end of culture. When the concentration of d-xylose fell below 20 g L^−1^, 70 g L^−1^ d-xylose was added. Samples were taken to monitor cell density, residual sugar, and organic acid accumulation.

### Fluorescence intensity characterization

*E. coli* JM109 cells carrying corresponding plasmids were grown in 5 mL LB plus antibiotics at 37 °C for 12 h in an Innova 44 shaker (Eppendorf, Germany) at 200 r.p.m. The culture was diluted 1:100 in 200 μL of fresh LB plus antibiotics and grown at 30 °C for 3 h with vigorous shaking at 1000 r.p.m. in a Titramax 1000 incubator (Heidolph Instruments, Germany). For time-course measurements, this was the *t* = 0 h time point. To maintain the cells in exponential growth phase, the culture was diluted 1:5-fold every 2 h. Samples were taken as required and were further diluted 1:10 in 200 μL of fresh LB with antibiotics and inducers for measurement using a SpectraMax M3 microplate reader (Molecular Devices, USA). GFP abundance was quantified at an excitation wavelength of 488 ± 10 nm and an emission wavelength of 511 ± 10 nm. YFP abundance was quantified at an excitation wavelength of 515 ± 10 nm and an emission wavelength of 544 ± 10 nm. mCherry abundance was quantified at an excitation wavelength of 588 ± 10 nm and an emission wavelength of 644 ± 10 nm.

For oscillation analysis, strains were cultured in LB medium in 250 mL shaker flasks at 30 °C, 200 r.p.m. When OD_600_ reached 0.6, 0.5 mM IPTG and 200 ng mL^−1^ ATC were added. For time-course determinations, this was the *t* = 0 h time point. Continuous sampling was performed for fluorescence detection on a SpectraMax M3 microplate reader.

For flow cytometry analysis, sample cells were washed twice with phosphate-buffered saline (PBS) and resuspended to an OD_600_ of 0.2. The assays were performed using a LSR Fortessa instrument (BD Biosciences) using fluorescein isothiocyanate (FITC) (GFP) and PE-TxRed (mCherry) channels. The voltage gains for each detector were set to FITC, 407 V and PE-TxRed, 650 V. Compensation was performed using cells that express only GFP or mCherry. For each sample, at least 10,000 counts were recorded using a 0.5 mL s^−1^ flow rate. All data were exported in FCS3 format and processed using FlowJo software (FlowJo, LLC).

### Single-cell time-lapse fluorescence microscopy

The molten LB medium was poured onto a glass slide after adding appropriate antibiotics (Amp and Cm) and inducers (IPTG and ATC). This agarose pad was solidified at room temperature. When the density of exponentially growing strains reached 0.8, inducers of IPTG and ATC were added. Then, 1 μL of cultures were pipetted onto an agarose pad and a cover slide was put on the top softly to prevent evaporation. Strain was in situ cultured at 30 °C and this time was set as zero-time point. Microscopy images were taken using a Nikon ECLIPSE 80i microscope equipped with a ×100 oil-immersion objective. Phase-contrast and fluorescence time-lapse images were recorded every 30 min using a Nikon DS-Ri1 camera. Bright-field images (intensity, 40%; exposure, 30 ms) and GFP fluorescence images (intensity, 80%; excitation, 495 nm; emission, 525/50 nm, exposure, 600 ms) were analyzed using ImageJ software.

### Kinetics experiment

For testing the effects of inducing TEVp on the degradation of fluorescent proteins, cultures were first grown in the pre-induction condition in shaker flasks (LB + 0.4% glucose, 0.5 mM IPTG), where target mCherry protein was expressed. Upon the OD_600_ reaching 0.3 (2 h), 1 mL of culture was sampled, centrifuged at 5000 × *g* for 5 min, and resuspended in PBS (pH 7.4, 37 °C). Samples were centrifuged again under the same conditions and then inoculated into a fresh shaker flask with LB media with no IPTG but 200 ng mL^−1^ ATC.

For testing the effects of inducing TEVp on the accumulation of fluorescent proteins, cultures were first grown in LB media in shaker flasks overnight. Then, cells were inoculated into 250 mL shaker flasks (working volume of 25 mL) at a dilution of 1:100 in LB media (with 200 ng mL^−1^ ATC and 0.5 mM IPTG). The fluorescence and OD_600_ at the zero-time point was determined by extrapolating a negative control condition (no TEVp expression) back to *t* = 0 h.

### SHK kinase activity assay

Strains were cultured in NBS medium and collected every 6 or 12 h, centrifuged at 12,000 × *g* for 10 min, washed with cold saline solution, and then resuspended in 0.05 M barbital buffer (pH 7.0). Crude extracts were lysed by sonication and centrifuged at 12,000 × *g* for 10 min. Protein concentration was determined by the Bradford method using bovine serum albumin as a standard. SHKkinase activity^51^ was measured in a 1 mL reaction mixture containing 4 μM ATP, 1 μM SHK, 10 μM NaF, 5 μM MgCl_2_, 25 μM barbital buffer (pH 9.0), and cell extract with 0.1–1.0 mg of protein. One unit of SHK kinase corresponded to 1 μM of SHK consumed per minute.

### Strain vitality and viability test

For vitality staining, 100 ng mL^−1^ propidium iodide (catalog number P3556; Invitrogen) was added to diluted samples and incubated for 15 min. Images were acquired using a Nikon ECLIPSE 80i microscope equipped with a Nikon DS-Ri1 camera. The viability of cells was assessed by inoculating LB plates with cells grown in liquid NBS. Cells were diluted to OD_600_ = 0.5 and 10 μL of 10× serially diluted cell suspension was spread on each agar plate. Plates were incubated at 37 °C for 12 h before counting.

### pHluorin calibration

Cells were incubated on ice for 5 min, centrifuged, and resuspended with cold PBS buffer. The collected cells were incubated at 37 °C for 30 min with solutions containing 150 mM KCl, 20 μM nigericin, and 50 mM buffering agents (for pHs ≤ 5.5, sodium acetate; for pHs 6 to 6.5, morpholine-ethane-sulfonic acid; for pHs 7 to 8, phosphate). The ratio (*R*_410/470_ values) of pHluorin fluorescence emitted (510 nm) under excitation at 410 and 470 nm was used to measure intracellular pH.

### Analysis of chemical concentrations

The concentrations of SHK, acetate, glucose, d-xylose, and d-xylonate were quantified with an HPLC system (Agilent 1260 Infinity, USA), equipped with an Aminex HPX-87H ion-exchange column (300 × 7.8 mm, Bio-Rad, USA) and a refractive index detector. Analysis was performed with a mobile phase of 5 mM sulfuric acid (65 °C) at a flow rate of 0.6 mL min^−1^ and detected by monitoring absorbance at 210 nm.

### Reporting summary

Further information on research design is available in the Nature Research Reporting Summary linked to this article.

## Supplementary information

Supplementary Information

Reporting Summary

Description of Additional Supplementary Files

Supplementary Data 1

Source Data

## Data Availability

Data supporting the findings of this work are available within the paper and its Supplementary Information files. A reporting summary for this Article is available as a Supplementary Information file. The datasets generated and analyzed during the current study are available from the corresponding author upon request. The GenBank accession numbers and the associated hyperlinks of the 14 key plasmids developed in this study are provided in Supplementary Table 7. The source data underlying Fig. 1c–f, 1i, 1j, 2b–e, 3c–d, 4, 5b–g, 6b–d, and 7b–e, as well as Supplementary Figure 1b, 2, 3c, 4d–e, 6b, 7b, 8, 9a–b, 11, 12b, 13, 15–19 are provided in Source Data file.
