# Supplementary material for: Programmable biomolecular switches for rewiring flux in Escherichia coli
Source: Nat Commun. 2019 Aug 21;10:3751. doi: 10.1038/s41467-019-11793-7 (PMC6704175; doi:10.1038/s41467-019-11793-7)
Supplement: Supplementary file 1 — Supplementary Information [file 41467_2019_11793_MOESM1_ESM.pdf]

Programmable biomolecular switches for rewiring flux in *Escherichia coli*

Gao *et al.*

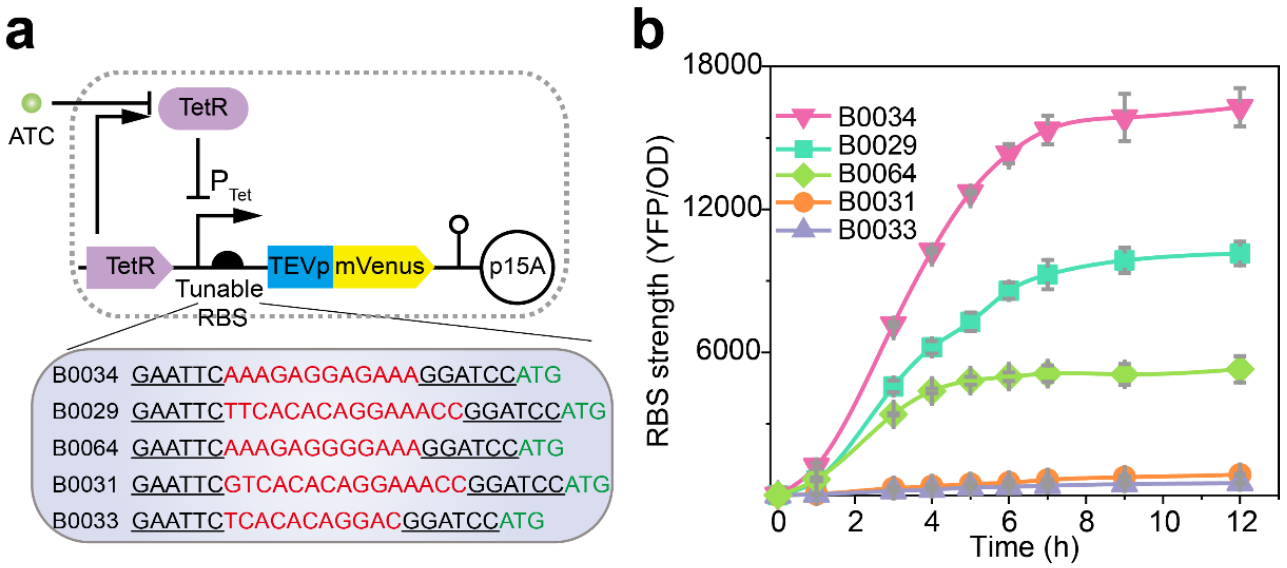

Supplementary Figure 1. RBS strength analysis. (a) Selected RBSs are taken from the MIT Registry of Standard Biological Parts. The red sequence is RBS core sequence. Underscored sequence shows *Eco*RI and *Bam*HI site. Start codon is shown in green color. **(b)** The characterization of promoter activity using five versions of RBS harbored by *E. coli* BL21(DE3) in LB medium at 30°C. Values are shown as mean ± s.d. from three (n = 3) biological replicates. Source data of Supplementary Figure 1b are provided in Source Data file.

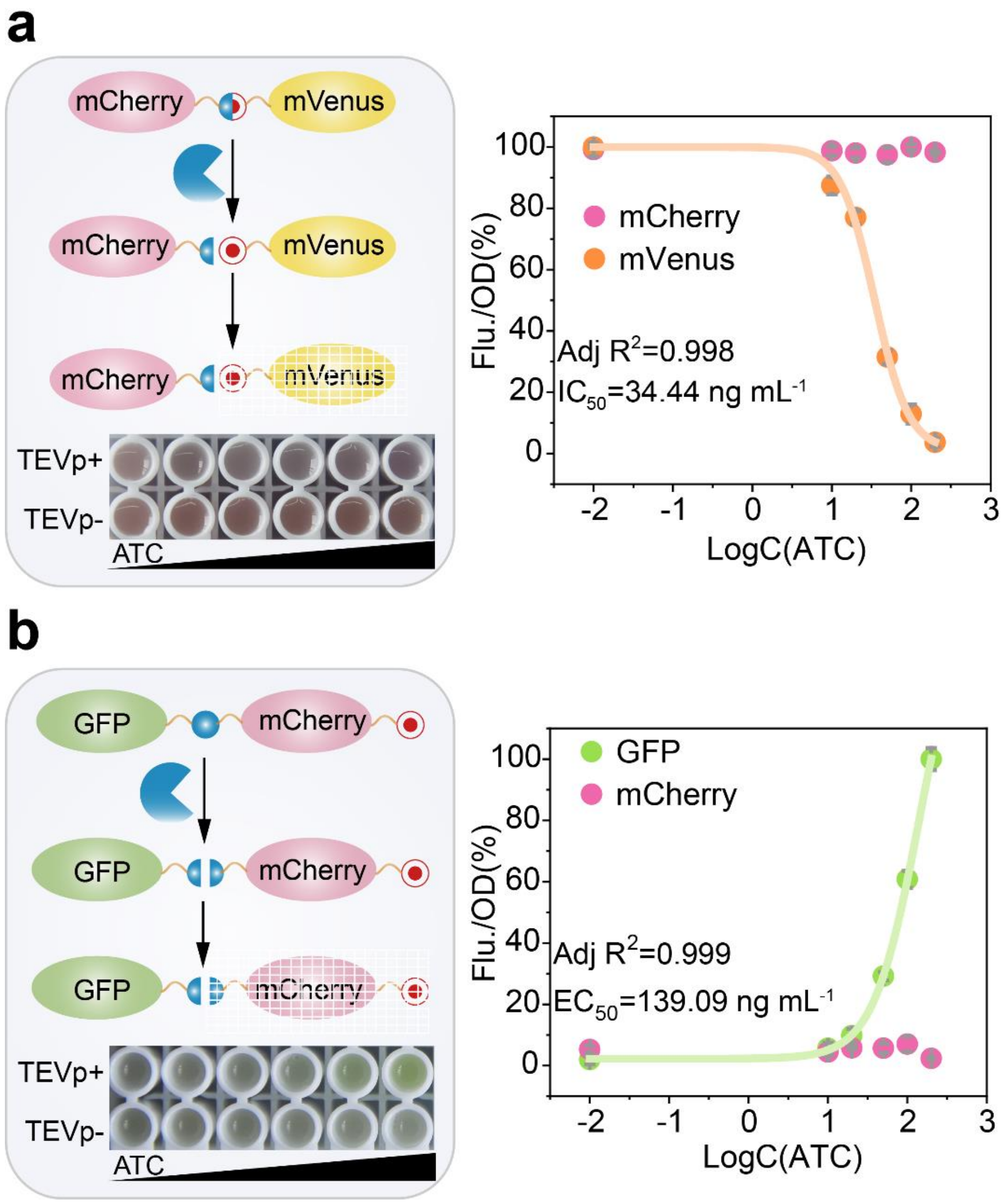

Supplementary Figure 2. Specificity of ON/OFF-switch regulatory unit. (a) Schematic of introducing nontarget protein mCherry in OFF-switch regulatory unit and dose-response curve of OFF-switch unit. Non-linear DoseResp model was used to fit the data shown by the orange lines. **(b)** Schematic of introducing nontarget protein mCherry in ON-switch regulatory unit and dose-response curve of ON-switch unit. Non-linear DoseResp model was used to fit the data shown by the green lines. Photograph of samples in the microwell plate from the 24 h time point following centrifugation and resuspension (200 μL). Values are shown as mean ± s.d. from three (n = 3) biological replicates. Specific anhydrotetracycline (ATC) concentration were 0, 5, 10, 50, 100, 200 ng mL^{-1}. Source data are provided in Source Data file.

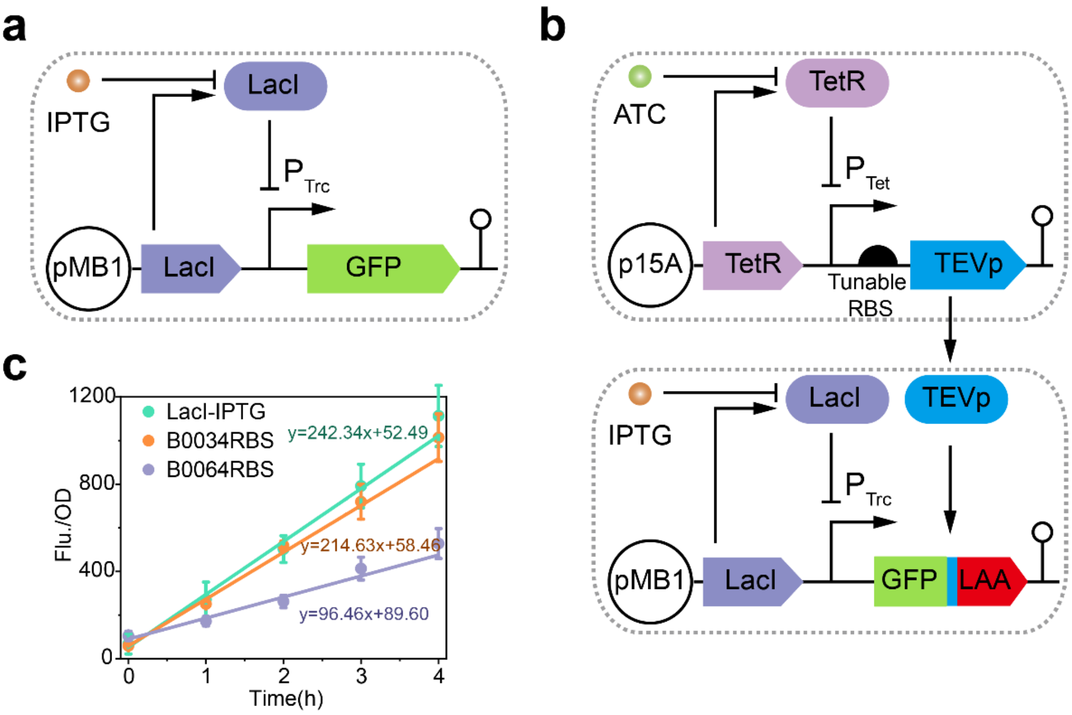

Supplementary Figure 3. Kinetic comparison between the ON-switch unit regulation system and LacI-IPTG inducible system. (a) Schematic of LacI-IPTG inducible system. **(b)** Schematic of ON-switch unit. **(c)** Kinetic comparison between two approaches. Kinetic of two approaches was benchmarked using a GFP reporter plasmid with promoter P_{Trc}, the time of adding inducer was defined as zero. Two kinds of ON-switch units with higher (B0034 RBS) and middle (B0064 RBS) TEVp level were tested by adding 200 ng mL^{-1} ATC at exponential growth period (OD_{600}= 0.6). Values are shown as mean ± s.d. from three (n = 3) biological replicates. Source data of Supplementary Figure 3c are provided in Source Data file.

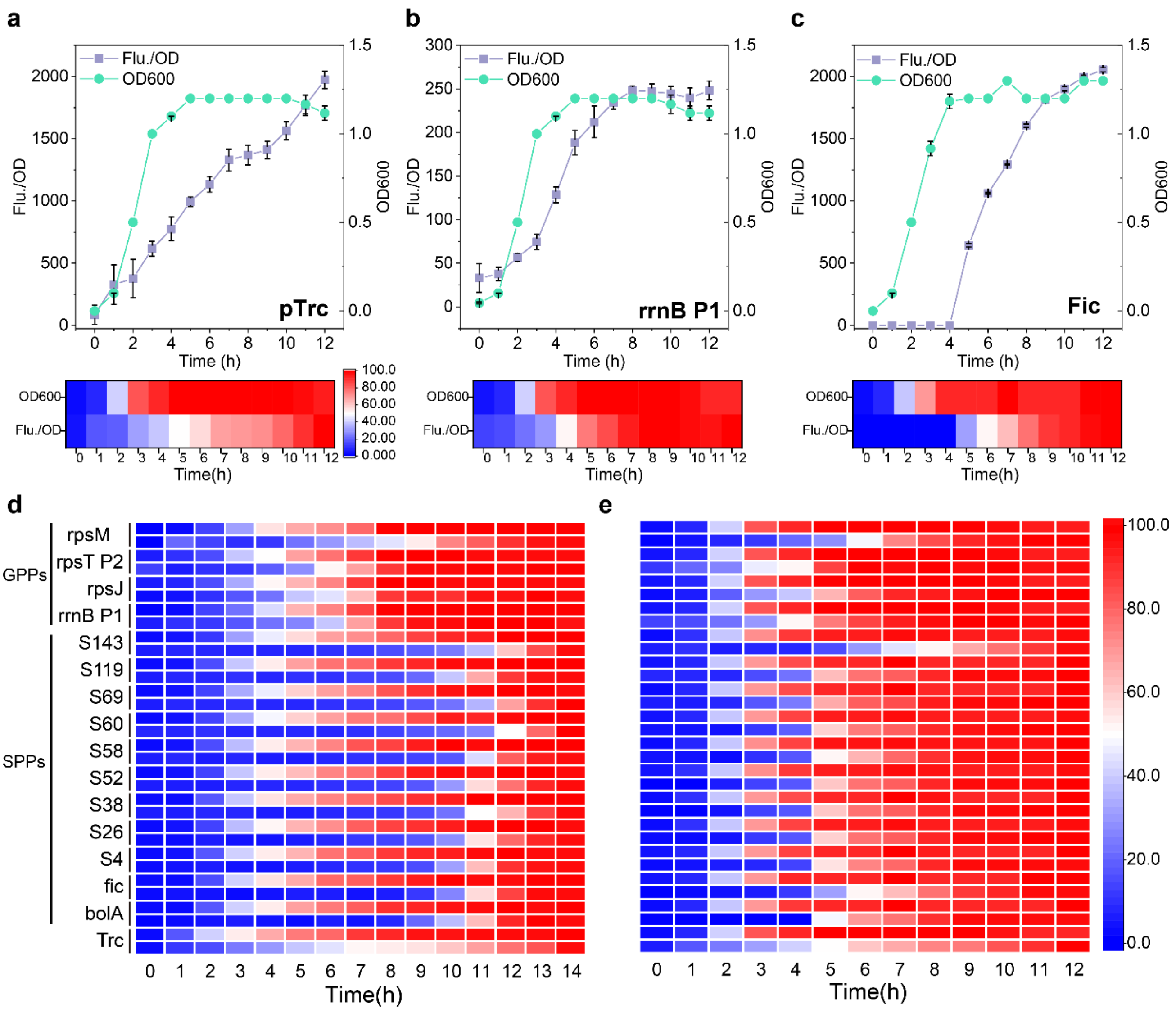

Supplementary Figure 4. Characterization of GPPs and SPPs. (a, b, c) Bacterial growth and specific fluorescence of *E. coli* MG 1655 containing the mVenus gene expressed by constitutive P_{Trc} promoter (a), growth phase promoter $P_{rrnB\ P1}$ (b), and stationary phase promoter P_{fic} (c) in LB medium at 30°C. **(d)** Characterization of different promoters on mVenus protein accumulation and cell growth in LB medium at 30°C. **(e)** Characterization of different promoters on mVenus protein accumulation and cell growth on 37°C. Color indicates the percent level. The highest value of cell density and fluorescence was defined as 100%. Each strain has two rows of squares in which the above one represents cell density, and the below one stands for mVenus protein abundance. Values are shown as mean ± s.d. from three biological replicates. Source data of Supplementary Figure 4d and 4e are provided in Source Data file.

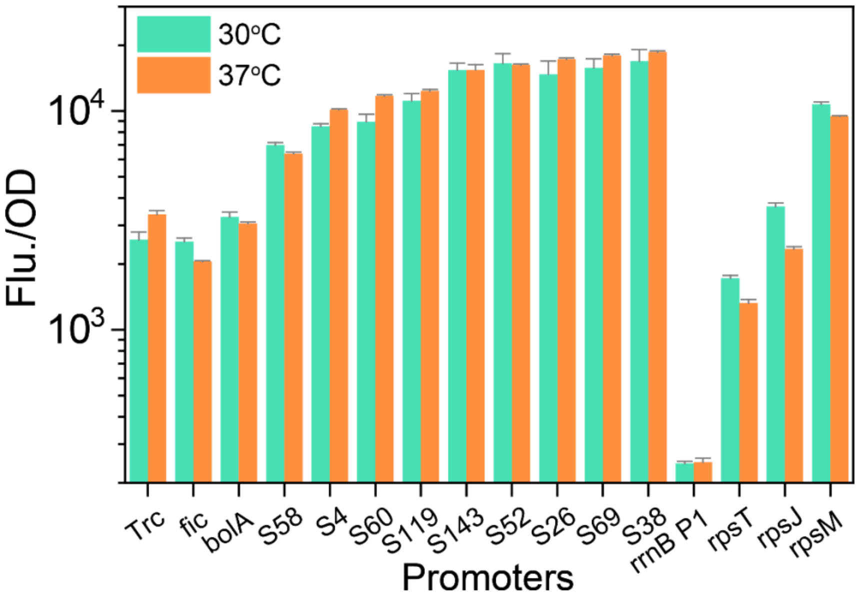

Supplementary Figure 5. Promoter activities of different promoters on mVenus protein expression. *E. coli* harboring reporter plasmid was cultured in LB medium at 37°C. Fluorescence intensity values were obtained at 12 h. Values are shown as mean ± s.d. from three biological replicates.

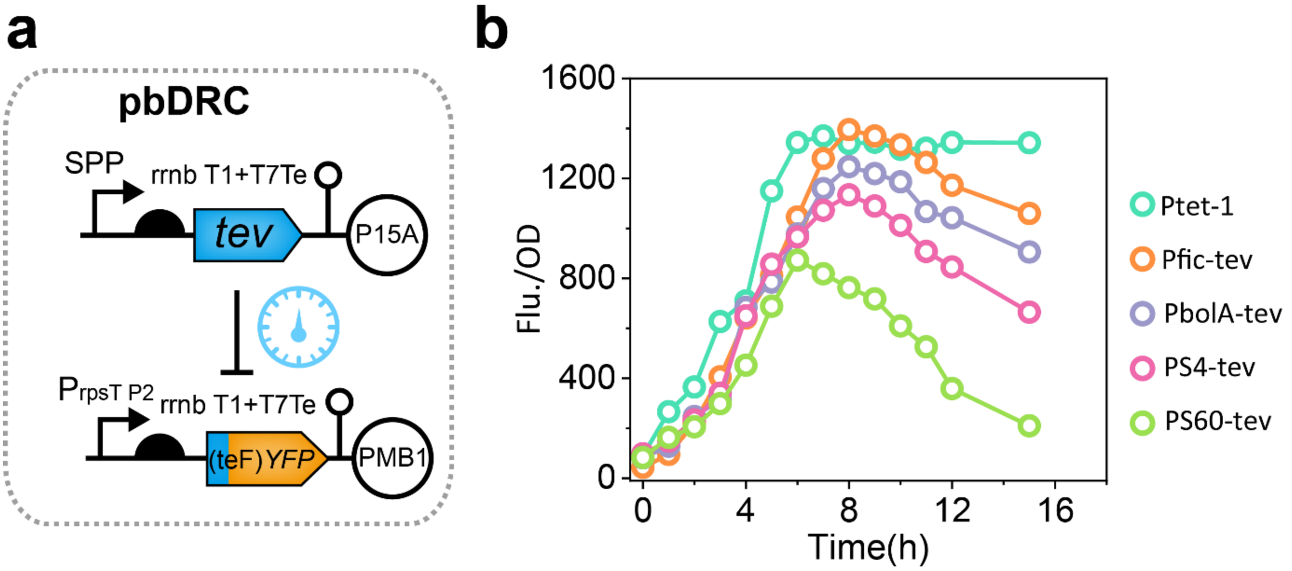

Supplementary Figure 6. Characterization of pbDRC. (a) Schematic of a pbDRC design. The time difference in the initiation of gene transcription by different physiological-dependent promoters provide prescribed switch time in controlling protein abundance. **(b)** The fluorescence abundance curve of strains with promoter $P_{rpsT\ P2}$-driven degradable YFP and SPPs-driven TEVp. Ptet-1 represented a control plasmid that without expression of TEVp. Source data of Supplementary Figure 6b are provided in Source Data file.

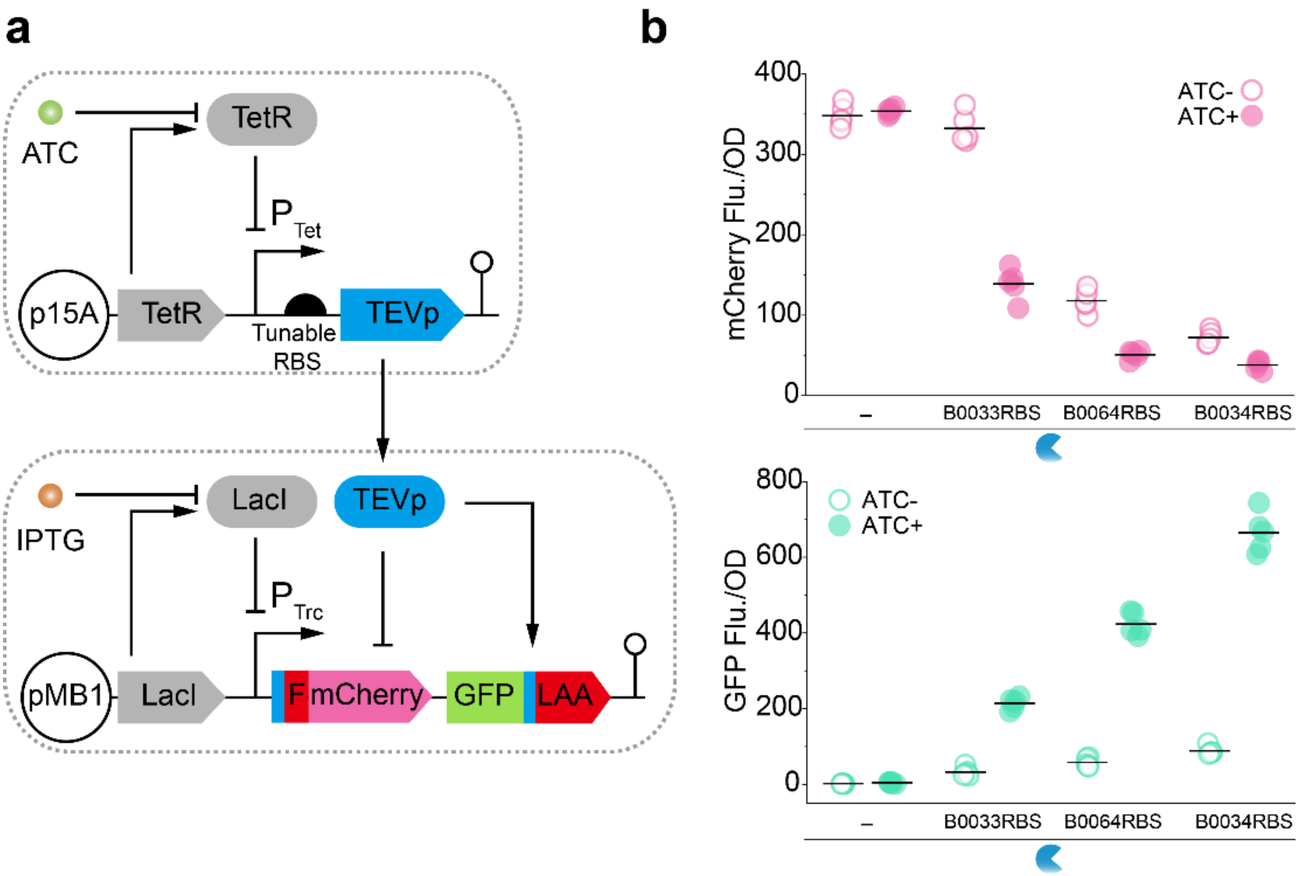

Supplementary Figure 7. Characterization of the one protease-based inverter. (a) System design. **(b)** GFP and mCherry abundance tuning with different TEVp expression strength. In all panels, the dark line indicates the mean values from six parallel samples. Error bars mean ± s. e. Source data of Supplementary Figure 7b are provided in Source Data file.

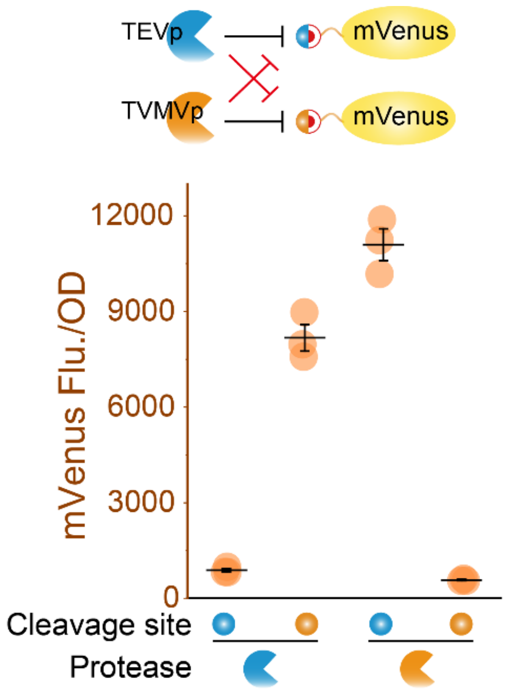

Supplementary Figure 8. The orthogonal cleave test of TEVp and TVMVp. The dark line indicates the mean values from six parallel samples. Error bars mean ± s. e. Source data are provided in Source Data file.

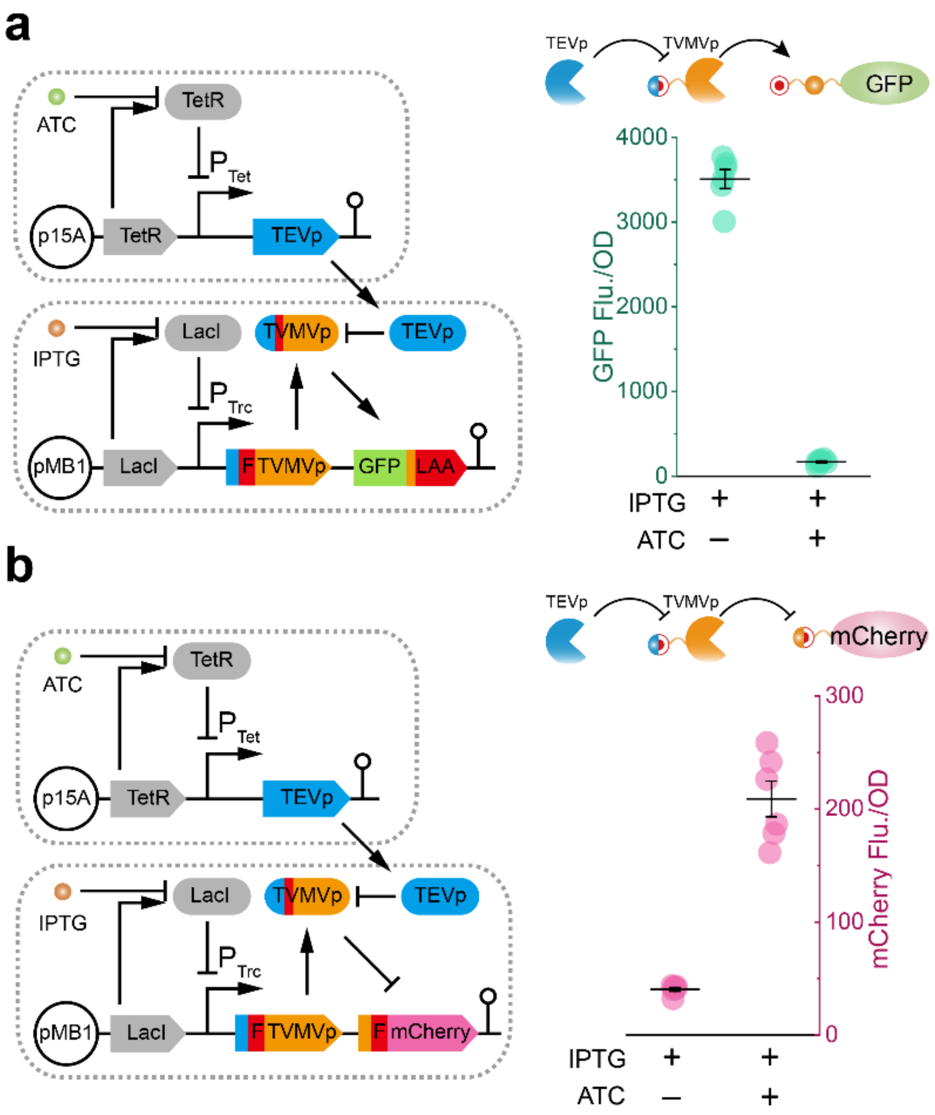

Supplementary Figure 9. Characterization of the protease-based inverter. (a) Layered protease cascade design. TVMVp-activated GFP could be repressed by introducing TVMVp-cleavable TEVp. **(b)** On the other hand, TVMVp-repressed mCherry protein could be activated by introducing TVMVp-cleavable TEVp. In all panels, the dark line indicates the mean values from six parallel samples. Error bars mean ± s. e. Source data are provided in Source Data file.

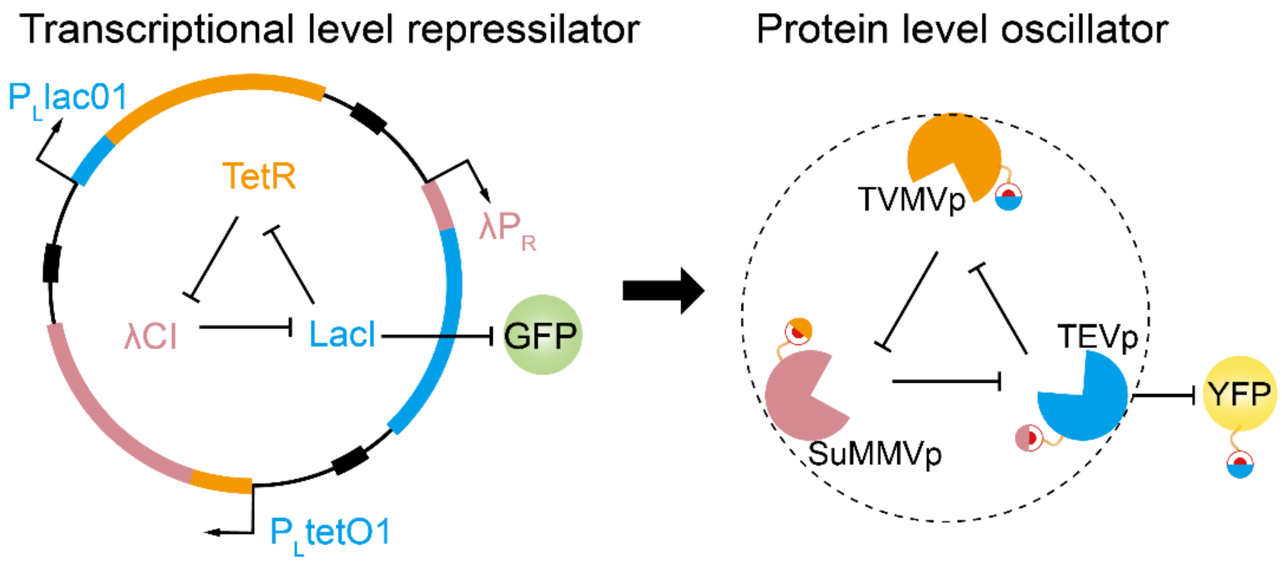

Supplementary Figure 10. The design inspiration of protein-level oscillator. Left: oscillation in transcriptional level repressilator is achieved by three orthogonal repressor-promoter pairs. Right: each protease was modified by fusing their N-terminus with a degron and other protease cleavage sites that could be specifically recognized and degraded by corresponding proteases.

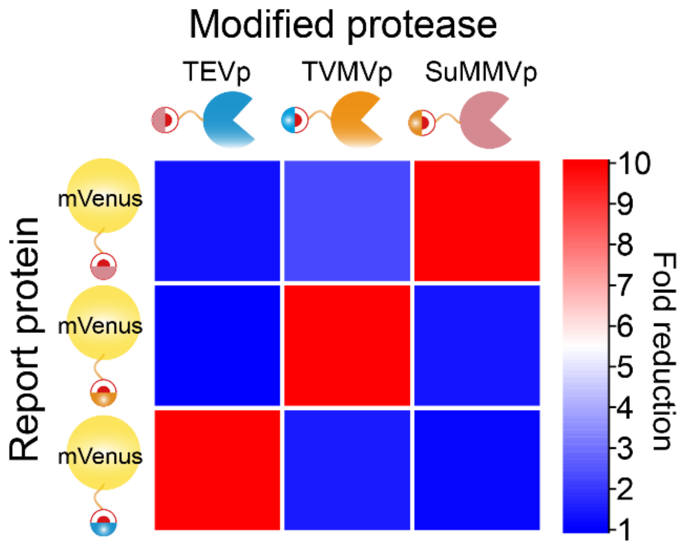

Supplementary Figure 11. Orthogonality matrix of modified protease and corresponding reporter. Values are shown as mean ± s.d. from three (n = 3) biological replicates. Source data are provided in Source Data file.

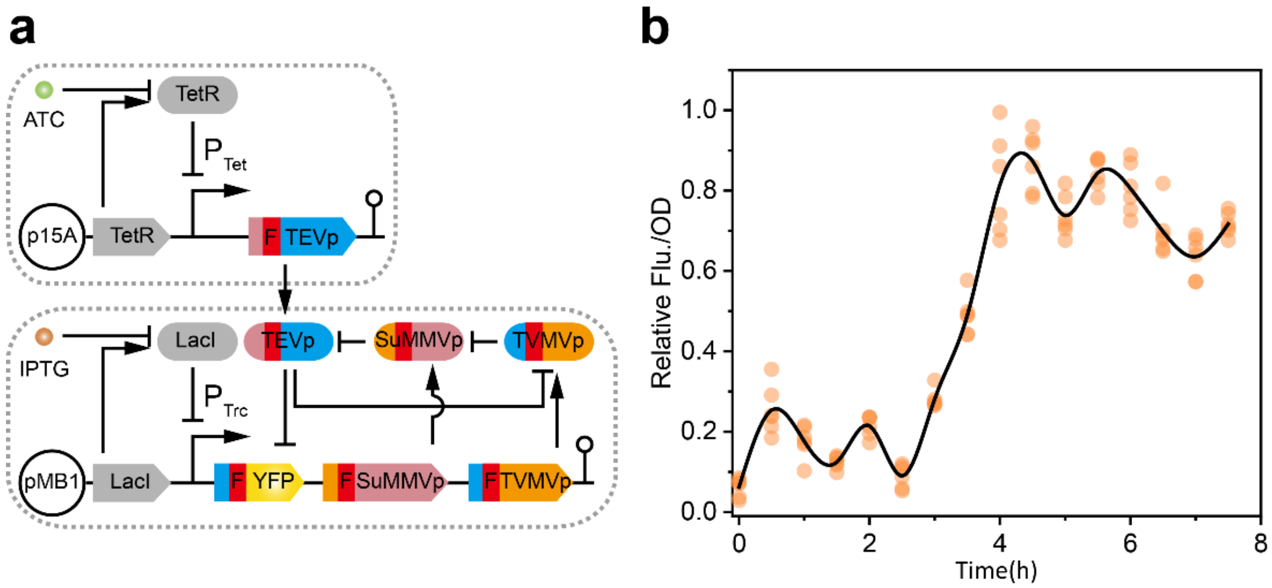

Supplementary Figure 12. Protease levels affect the stability of the oscillator system. (a) One kind of protein oscillator design. TEVp was expressed on a low copy number trigger plasmid (p15A ori), while other two proteases TVMVp and SuMMVp were co-expressed with the reporter mVenus (YFP) on a high copy number plasmid (pMB1 ori). **(b)** Characterization of fluorescence curve. Black trace was the mean value taken from six separate colonies across the array. Error bars mean ± s. e. Source data of Supplementary Figure 12b are provided in Source Data file.

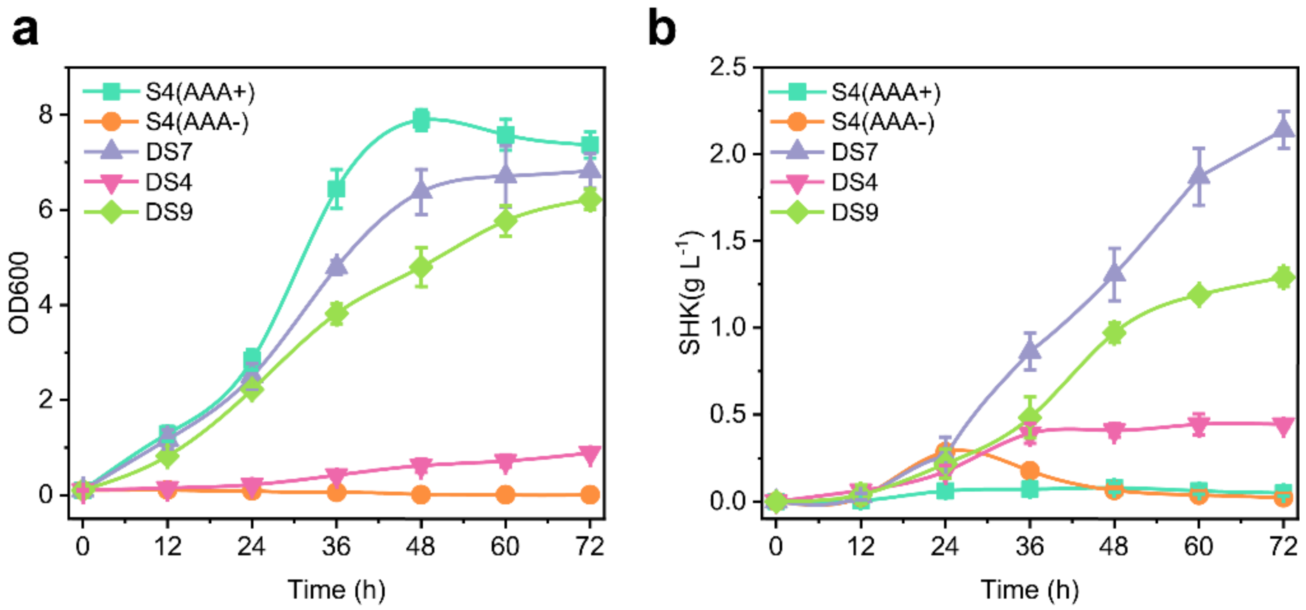

Supplementary Figure 13. Cell growth and shikimate production curves of typical strains. (a) Cell growth curve of four kinds of strains (Strain DS7, DS4, DS9 represented three kinds of variants equipped with pbDRC cultured in NBS medium without AAA supplement; chassis S4 cultured in NBS medium with (+) or without (-) AAA supplement was set as control strain). **(b)** Shikimate production curves of corresponding strains. All the data was obtained at 33°C, 200 rpm. Values are shown as mean ± s.d. from three (n = 3) biological replicates. Source data are provided in Source Data file.

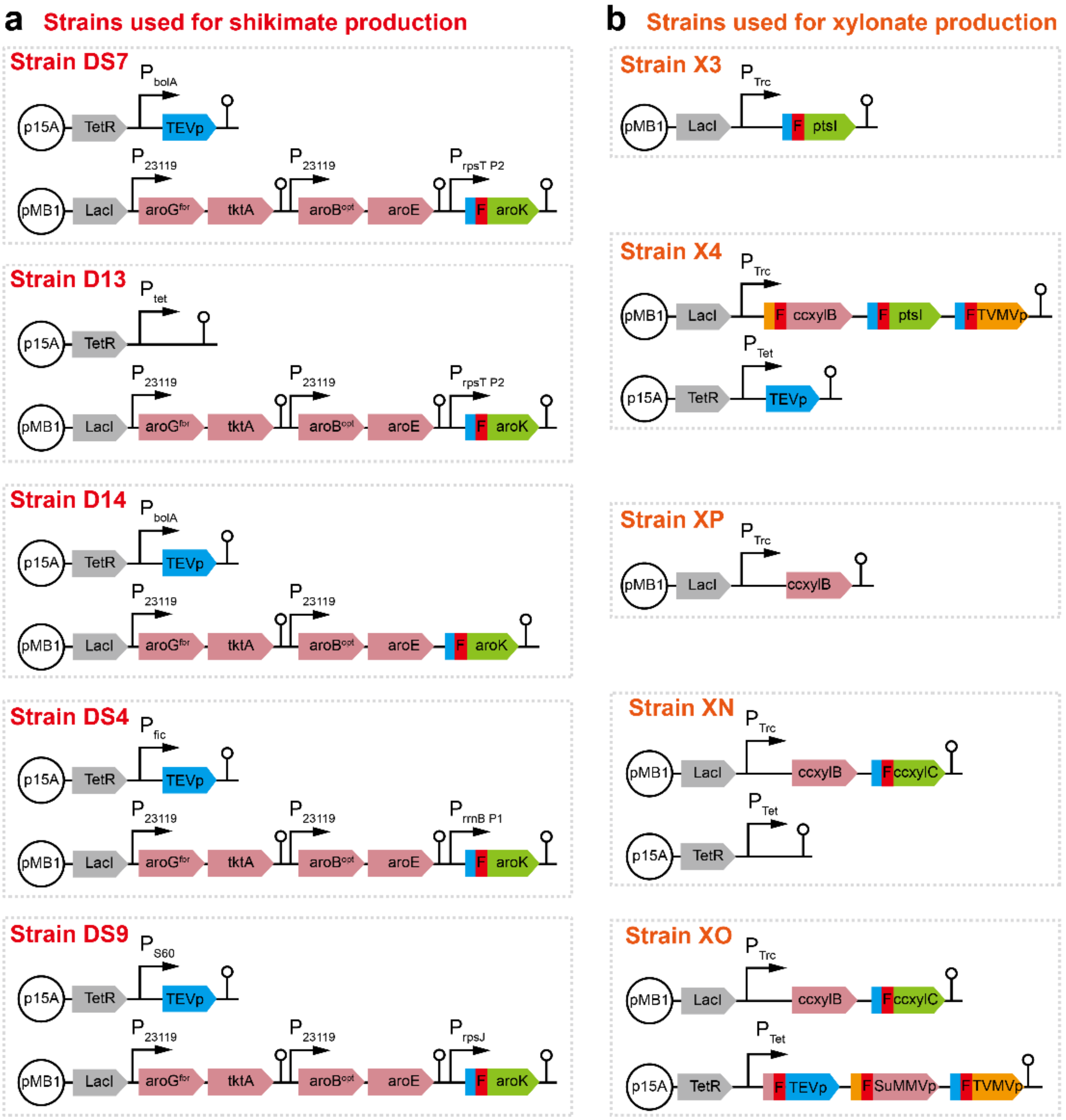

Supplementary Figure 14. Schematic of plasmid constructs in strain variants used for shikimate and xylonate production. (a) shikimate production. (b) xylonate production.

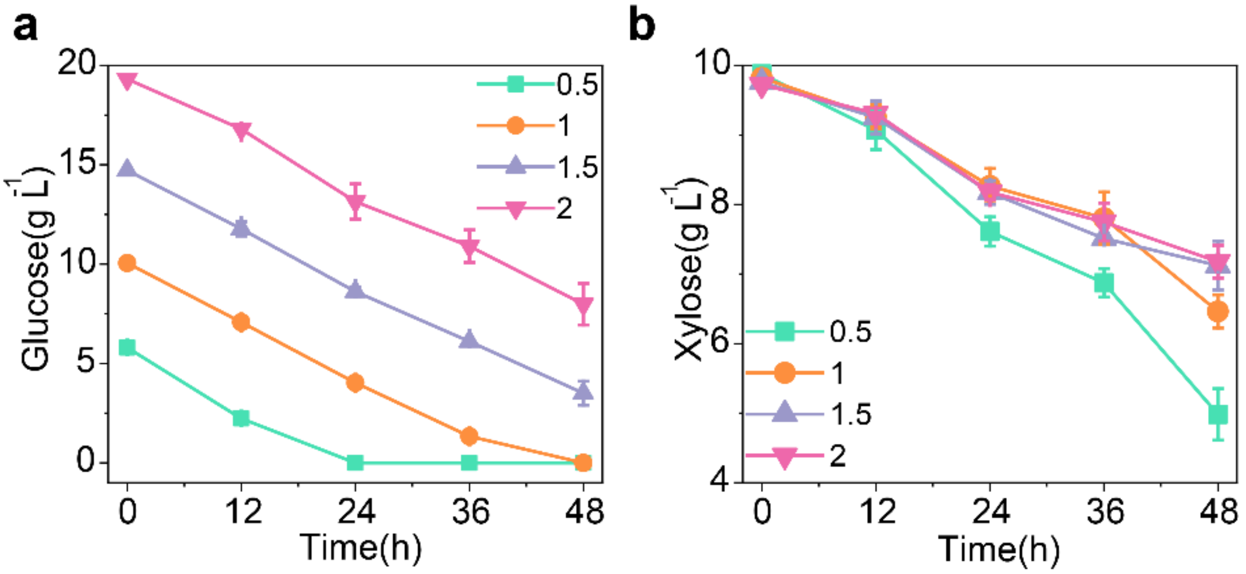

Supplementary Figure 15. Effect of carbon catabolite repression in mix sugar fermentation. (**a**) The glucose consumption curve of strain X1 with different sugar ratios (0.5, 5 g L^{-1} glucose + 10 g L^{-1} xylose; 1, 10 g L^{-1} glucose + 10 g L^{-1} xylose; 1.5, 15 g L^{-1} glucose + 10 g L^{-1} xylose; 2, 20 g L^{-1} glucose + 10 g L^{-1} xylose). (**b**) The xylose consumption curve of strain X1 with different sugar ratios. All the data was obtained using NBS minimal medium at 37°C, 200 rpm. Values are shown as mean ± s.d. from three (n = 3) biological replicates. Source data are provided in Source Data file.

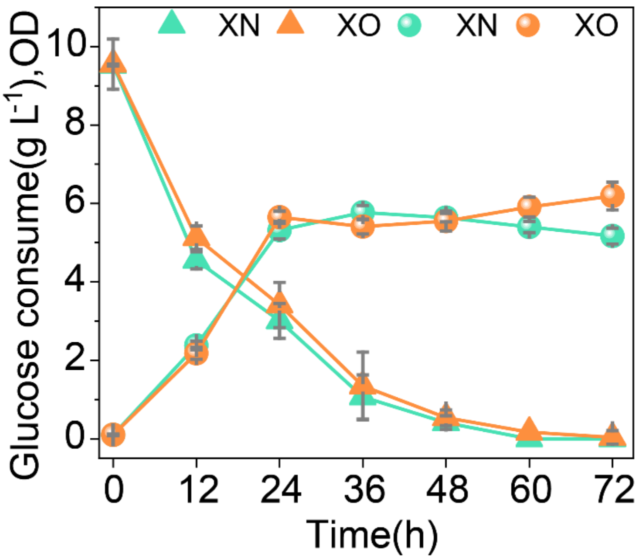

Supplementary Figure 16. Time course of strain XN and XO in glucose consumption and cell density. Glucose consumption: solid triangles; cell density: soild circles. Values are shown as mean ± s.d. (n=3). Source data are provided in Source Data file.

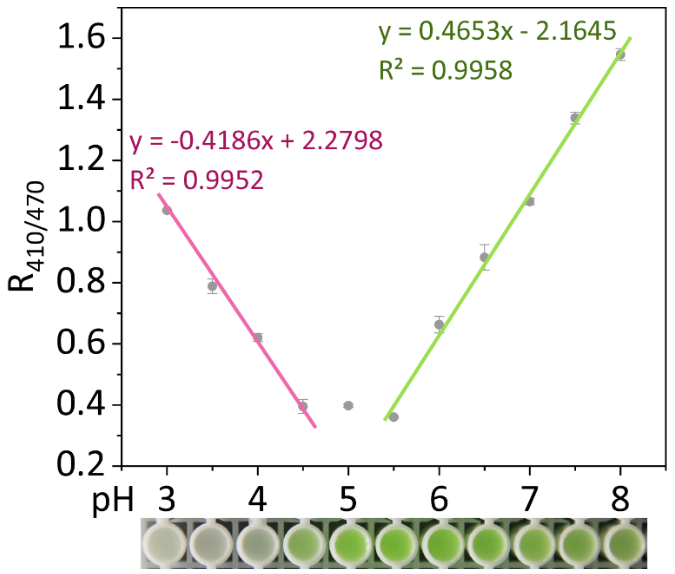

Supplementary Figure 17. The biphasic linear correlation between cytoplasm pH value and $R_{410/470}$ value. Microwell plate photograph of permeabilized cells after 1 h treatment was also provided below (300 μL). Values are means of three biological replicates. Source data are provided in Source Data file.

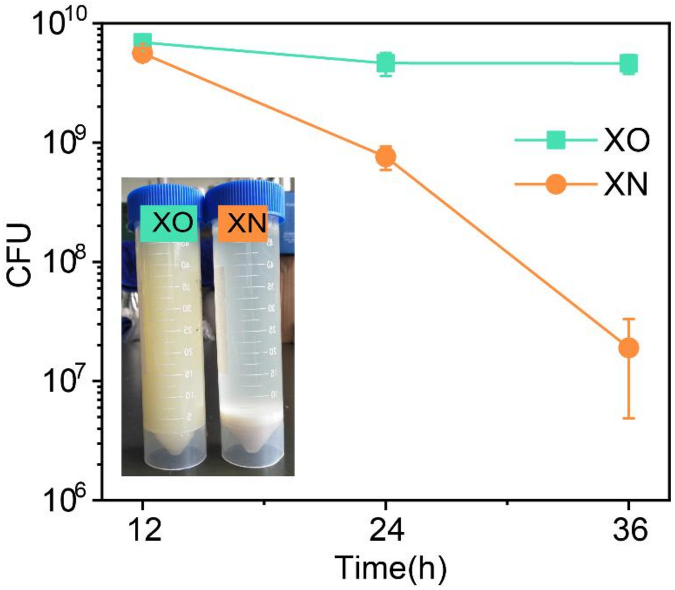

Supplementary Figure 18. Cell viability of different strains during xylonate production. Insert: photograph of cells in D-xylonate production (72 h) after 1 h natural precipitation. Values are shown as mean ± s.d. from three (n = 3) biological replicates. Source data are provided in Source Data file.

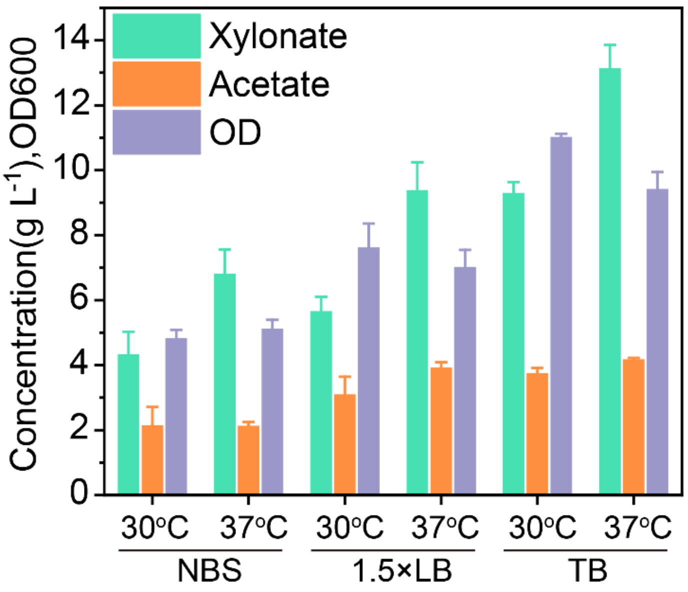

Supplementary Figure 19. Fermentation conditions optimization. Six fermentation conditions including medium (NBS, 1.5 × LB, TB) and temperature (30°C, 37°C) were implemented for boosting D-xylonate titre. Data was obtained at the end point of fermentation (48 h). Values are shown as mean ± s.d. from three (n = 3) biological replicates. Source data are provided in Source Data file.

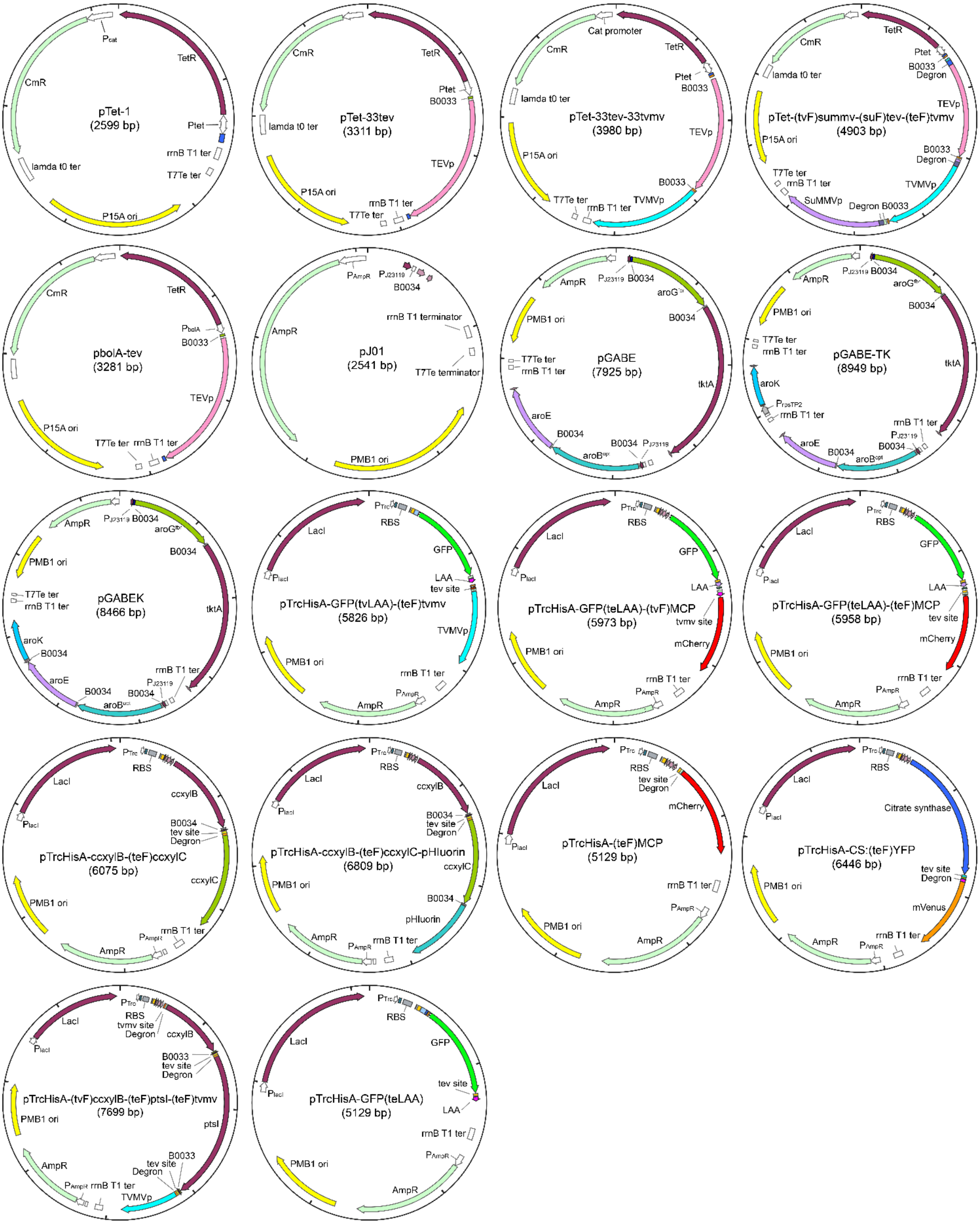

Supplementary Figure 20. Map of key plasmids used in this study. Drawing by SnapGene software.

Supplementary Table 1. Primers used in this study.

Name	Sequence(5'-3')	Purpose
YZ-tet-F	ccttcgattccgacctcat	DNA fragment inserting confirmation
YZ-tet-R	gttcaccgacaaacaacagata	DNA fragment inserting confirmation
YZ-PJ01-F	aaaataggcgtatcacgaggca	DNA fragment inserting confirmation
YZ-PJ01-R	gcgttcaccgacaaacaacaga	DNA fragment inserting confirmation
KZ-AG1-U	agtaaagaggagaaaaagcttggatgaattatcagaacgacgatttac	Site-directed mutagenesis (*aroG^{fbr}*)
KZ-AG1-D	attgagaaactcacctgccg	Site-directed mutagenesis (*aroG^{fbr}*)
KZ-AG2-U	cggcaggtgagtttctcaatatgat	Site-directed mutagenesis (*aroG^{fbr}*)
KZ-AG2-D	cctctttaccagtttacccgcgacgcgcttt	Site-directed mutagenesis (*aroG^{fbr}*)
KZ-TK-U	cgggtaaactggtaaagaggagaaaaagcttggatgtcctcacgtaaagagcttg	*tktA* cloning in pJ01
KZ-TK-D	atgatgatgatgatggtcgacttacagcagttcttttgctttc	*tktA* cloning in pJ01
KZ-AB-U	agtaaagaggagaaaaagcttatggagcgtattgtcgttactct	*aroB^{opt}* cloning in pJ01
KZ-AB-D	taggcctgtcctgtgtgagagctcttacgctgattgacaatcggc	*aroB^{opt}* cloning in pJ01
KZ-AE-U	tgtcaatcagcgtaagagctcaaagaggagaaaaagcttatggaaacctatgctgtttttgg	*aroE* cloning in pJ01
KZ-AE-D	atgatgatgatgatggtcgactcacgcggacaattcctcc	*aroE* cloning in pJ01
KZ-(su)tev-F	ttcttattcgtgcaagaactgggtgaaagcctgtttaaaggt	*Tev* cloning in pTet-1
KZ-(su)tev-R	ctgcagatgaatttcttcgctacccggatcctggctgtggt	*Tev* cloning in pTet-1
KZ-(te)tvmv-F	cgggatcctggtagcgaaaacctgtattttcagttcttattcgtgcaagaactgagtaaagccctgctgaaag	*Tvmv* cloning in pTet-1
KZ-(te)tvmv-R	cccaagcttttattcaaccagggtaaaactgc	*Tvmv* cloning in pTet-1
KZ-(tv)summv-F	gcgagaccgtgcgcttccagtttttatttgtgcaagaactgggagtcagtcttagtcgtggg	*Summv* cloning in pTet-1
KZ-(tv)summv-R	cccaagcttttactgcactttaataccttccgta	*Summv* cloning in pTet-1
SG-aroK-F	agagaaacgcaatatctttcgttttagagctagaaatagcaag	SgRNA construction for *aroK* deletion
SG-aroL-F	ccatccaccgttatcgctacgttttagagctagaaatagcaagt	SgRNA construction for *aroL* deletion
SG-ptsI-F	ggtacggttcgtgacgttgagttttagagctagaaatagc	SgRNA construction for *PTS* deletion
sgRNA-R	actagtattatacctaggactgagc	SgRNA construction
U-aroK-F	gataaggatcgatggggatcccattccctggttcgggca	DNA fragment assembly for *aroK* deletion
U-aroK-R	aagccagaatttttcggtactactaagactattcgttaa	DNA fragment assembly for *aroK* deletion
D-aroK-F	gtaccgaaaaattctggctttatatacactcgtctgc	DNA fragment assembly for *aroK* deletion
D-aroK-R	aaacagccaagcttcgaattcgggatggttgaccgcagtt	DNA fragment assembly for *aroK* deletion
U-aroL-F	gataaggatcgatggggatccagcgcgacacaatagaggattac	DNA fragment assembly for *aroL* deletion
U-aroL-R	agctgtcaattagccacgactacgttgcact	DNA fragment assembly for *aroL* deletion
D-aroL-F	ctgctgtaattttcgagcgcctatacttaacg	DNA fragment assembly for *aroL* deletion
D-aroL-R	aaacagccaagcttcgaattcattgttcatccaccttacttttctttc	DNA fragment assembly for *aroL* deletion
KZ-ccxylB-F	gataaggatcgatggggatccatgtcttctgctatctacccgtctc	*ccxylB* cloning in pTrcHisA
KZ-ccxylB-R	cgactttctcctctttgagctcttaacgccaaccagcgtcga	*ccxylB* cloning in pTrcHisA
KZ-ccxylC-F	atgaccgcccaagttacttgt	*ccxylC* cloning in pTrcHisA
KZ-ccxylC-R	ttaaaccagacgcacttcatgc	*ccxylC* cloning in pTrcHisA
KZ-PH-F	agaaatctagaatgagtaaaggagaag	pHIuorin cloning in pTrcHisA
KZ-PH-R	gccaagcttttatttgtatagttcatccatgcc	pHIuorin cloning in pTrcHisA

Supplementary Table 2. List of plasmids used in the basic protein regulation unit construction.

Plasmid name	Plasmid characteristics
pTet-1	P_{tet}, p15A ori, Cm^R, tetR
pTrcHisA	P_{Trc}, pBR322 ori, Amp^R, $LacI^q$
pJ01	P_{J23119}, pMB1 ori, Amp^R
pTet-34*tev*	TEVp expression with B0034RBS on pTet-1
pTet-29*tev*	TEVp expression with B0029RBS on pTet-1
pTet-64*tev*	TEVp expression with B0064RBS on pTet-1
pTet-31*tev*	TEVp expression with B0031RBS on pTet-1
pTet-33*tev*	TEVp expression with B0033RBS on pTet-1
pTet-34*tev*:YFP	Fused protein (TEVp and YFP) was inserted in pTet-1 with B0034RBS
pTet-29*tev*:YFP	Fused protein (TEVp and YFP) was inserted in pTet-1 with B0029RBS
pTet-64*tev*:YFP	Fused protein (TEVp and YFP) was inserted in pTet-1 with B0064RBS
pTet-31*tev*:YFP	Fused protein (TEVp and YFP) was inserted in pTet-1 with B0031RBS
pTet-33*tev*:YFP	Fused protein (TEVp and YFP) was inserted in pTet-1 with B0033RBS
pTrcHisA-(teF)MCP	Fused protein containing *tev* site, F degron, mCherry was inserted in pTrcHisA
pTrcHisA-GFP(teLAA)	Fused protein containing GFP, *tev* site, *SsrA* tag was inserted in pTrcHisA
pTrcHisA-mCherry(teF)YFP	Fused protein containing mCherry, *tev* site, F degron and YFP was inserted in pTrcHisA
pTrcHisA-GFP(te)MCP(LAA)	Fused protein containing GFP, *tev* site, F degron and mCherry-*SsrA* was inserted in pTrcHisA
pTrcHisA-GFP	GFP was inserted in pTrcHisA

Supplementary Table 3. List of plasmids used in the dynamic regulation circuit construction.

Plasmid name	Plasmid characteristics
ptrc-YFP	YFP expression with Trc promoter on pJ01
ptrc-(teF)YFP	Fused protein containing tev site, F degron and YFP was expressed under Trc promoter on pJ01
pbolA-YFP	YFP expression with bolA promoter, B0034RBS on pJ01
pfic-YFP	YFP expression with fic promoter, B0034RBS on pJ01
pS4-YFP	YFP expression with S4 promoter, B0034RBS on pJ01
pS26-YFP	YFP expression with S26 promoter, B0034RBS on pJ01
pS38-YFP	YFP expression with S38 promoter, B0034RBS on pJ01
pS52-YFP	YFP expression with S52 promoter, B0034RBS on pJ01
pS58-YFP	YFP expression with S58 promoter, B0034RBS on pJ01
pS60-YFP	YFP expression with S60 promoter, B0034RBS on pJ01
pS69-YFP	YFP expression with S69 promoter, B0034RBS on pJ01
pS119-YFP	YFP expression with S119 promoter, B0034RBS on pJ01
pS143-YFP	YFP expression with S143 promoter, B0034RBS on pJ01
prpsM-(teF)YFP	(teF)YFP expression with rpsM promoter, B0034RBS on pJ01
prpsT-(teF)YFP	(teF)YFP expression with rpsT P2 promoter, B0034RBS on pJ01
prpsJ-(teF)YFP	(teF)YFP expression with rpsJ promoter, B0034RBS on pJ01
prrnB-(teF)YFP	(teF)YFP expression with rrnB P1 promoter, B0034RBS on pJ01
pbolA-*tev*	TEVp expression with bolA promoter, B0034RBS on pTet-1
pfic-*tev*	TEVp expression with fic promoter, B0034RBS on pTet-1
pS4-*tev*	TEVp expression with S4 promoter, B0034RBS on pTet-1
pS60-*tev*	TEVp expression with S60 promoter, B0034RBS on pTet-1

Supplementary Table 4. List of plasmids used in the inverter construction.

Plasmid name	Plasmid characteristics
pTrcHisA-GFP(teLAA)-(teF)MCP	Fused protein (GFP, *tev* site, *SsrA* tag) and fused protein (*tev* site, F degron, mCherry) were inserted in pTrcHisA
pTrcHisA-GFP(teLAA)-(tvF)MCP	Fused protein (GFP, *tev* site, *SsrA* tag) and fused protein (*tvmv* site, F degron, mCherry) were inserted in pTrcHisA
pTrcHisA-CS:(tvF)YFP	Fused protein containing citrate synthase, *tvmv* site, F degron, HA and YFP was inserted in pTrcHisA
pTrcHisA-CS:(teF)YFP	Fused protein containing citrate synthase, *tev* site, F degron, HA and YFP was inserted in pTrcHisA
pTrcHisA-GFP(tvLAA)-(teF)*tvmv*	Fused protein (GFP, *tvmv* site, *SsrA* tag) and fused protein (*tev* site, F degron, *tvmv*) were inserted in pTrcHisA
pTrcHisA-(teF)*tvmv*-(tvF)MCP	Fused protein (*tev* site, F degron, *tvmv*) and fused protein (*tvmv* site, F degron, mCherry) were inserted in pTrcHisA
pTrcHisA-(teF)mCherry-(tvF)GFP-(teF)*tvmv*	Fused protein (*tev* site, F degron, *tvmv*), fused protein (*tvmv* site, F degron, GFP) and fused protein (*tev* site, F degron, mCherry) were co-expressed in pTrcHisA
pTet-33*tvmv*	TVMVp expression with B0033RBS on pTet-1
pTet-33*tev*-33*tvmv*	*Tev* with B0033RBS and *tvmv* with B0033RBS were co-expressed on pTet-1

Supplementary Table 5. List of plasmids used in the oscillator construction.

Plasmid name	Plasmid characteristics
pTrcHisA-CS:(suF)YFP	Fused protein containing citrate synthase, *summv* site, F degron, HA and YFP was inserted in pTrcHisA
pTrcHisA-(tvF)YFP-(tvF)*summv*-(teF)*tvmv*	Fused protein(*tvmv* site, F degron, YFP), fused protein (*tvmv* site, F degron, *summv*) and fused protein (*tev* site, F degron, *tvmv*) were inserted in pTrcHisA
pTet-*33summv*	SuMMVp expression with B0033RBS on pTet-1
pTet-(tvF)*summv*	Fused protein containing *tvmv* site, F degron, *summv* was expressed with B0033RBS on pTet-1
pTet-(suF)*tev*	Fused protein containing *summv* site, F degron, *tev* was expressed with B0033RBS on pTet-1
pTet-(teF)*tvmv*	Fused protein containing *tev* site, F degron, *tvmv* was expressed with B0033RBS on pTet-1
pTet-(tvF)*summv*-(suF)*tev*-(teF)*tvmv*	Fused protein (*tvmv* site, F degron, *summv*), fused protein (*summv* site, F degron, *tev*) and Fused protein (*tev* site, F degron, *tvmv*) were co-expressed in pTet-1

Supplementary Table 6. List of plasmids used for shikimate and D-xylonate production.

Plasmid name	Plasmid characteristics
pGABE	Gene *aroG*^{fbr}, *tktA*, *aroB*^{opt}, and *aroE* were co-expressed on pJ01
pGABEK	Gene *aroG*^{fbr}, *tktA*, *aroB*^{opt}, *aroE* and *aroK* were co-expressed on pJ01
pGABE-BK	pGABE, *aroK* expression with rrnB P1 promoter, B0034RBS on pJ01
pGABE-JK	pGABE, *aroK* expression with rpsJ promoter, B0034RBS on pJ01
pGABE-TK	pGABE, *aroK* expression with rpsT P2 promoter, B0034RBS on pJ01
pTrcHisA-*ccxylB*	Gene *ccxylB* was inserted in pTrcHisA
pTrcHisA-*ptsI*	Gene *ptsI* was inserted in pTrcHisA
pTrcHisA-(tvF)ccxylB-(teF)*ptsI*-(teF)*tvmv*	Fused protein (*tvmv* site, F degron, *ccxylB*), fused protein (*tev* site, F degron, *ptsI*) and fused protein (*tev* site, F degron, *tvmv*) were co-expressed in pTrcHisA
pTrcHisA-*ccxylB*-(teF)*ccxylC*	Gene *ccxylB* and fused protein (*tev* site, F degron, *ccxylC*) were co-expressed in pTrcHisA
pTrcHisA-pHluorin	Reporter pHluorin was inserted in pTrcHisA
pTrcHisA-*ccxylB*-pHluorin	Gene *ccxylB* and reporter pHluorin were inserted in pTrcHisA
pTrcHisA-*ccxylB*-(teF)*ccxylC*-pHluorin	Gene *ccxylB*, fused protein (*tev* site, F degron, *ccxylC*), and reporter pHluorin were co-expressed in pTrcHisA

Supplementary Table 7. The accession numbers of the key plasmids used in this study.

Plasmid name	GenBank accession number
pTet-1	MK234848 [https://www.ncbi.nlm.nih.gov/nuccore/MK234848]
pJ01	MK234843 [https://www.ncbi.nlm.nih.gov/nuccore/MK234843]
pTrcHisA-CS:(teF)YFP	MK238516 [https://www.ncbi.nlm.nih.gov/nuccore/MK238516]
pTet-33*tev*	MK238517 [https://www.ncbi.nlm.nih.gov/nuccore/MK238517]
pTrcHisA-(teF)MCP	MK238518 [https://www.ncbi.nlm.nih.gov/nuccore/MK238518]
pTrcHisA-GFP(teLAA)	MK238519 [https://www.ncbi.nlm.nih.gov/nuccore/MK238519]
pTrcHisA-GFP(teLAA)-(teF)MCP	MK258730 [https://www.ncbi.nlm.nih.gov/nuccore/MK258730]
pTrcHisA-GFP(tvLAA)-(teF)*tvmv*	MK258729 [https://www.ncbi.nlm.nih.gov/nuccore/MK258729]
pTet-33*tev*-33*tvmv*	MK258731 [https://www.ncbi.nlm.nih.gov/nuccore/MK258731]
pTet-(tvF)*summv*-(suF)*tev*-(teF)*tvmv*	MK238520 [https://www.ncbi.nlm.nih.gov/nuccore/MK238520]
pGABE	MK238521 [https://www.ncbi.nlm.nih.gov/nuccore/MK238521]
pTrcHisA-(tvF)*ccxylB*-(teF)*ptsI*-(teF)*tvmv*	MK258728 [https://www.ncbi.nlm.nih.gov/nuccore/MK258728]
pTrcHisA-*ccxylB*-(teF)*ccxylC*	MK258732 [https://www.ncbi.nlm.nih.gov/nuccore/MK258732]
pTrcHisA-*ccxylB*-(teF)*ccxylC*-pHluorin	MK258733 [https://www.ncbi.nlm.nih.gov/nuccore/MK258733]
