# Supplementary material for: Programmable biomolecular switches for rewiring flux in Escherichia coli
Source: Nat Commun. 2019 Aug 21;10:3751. doi: 10.1038/s41467-019-11793-7 (PMC6704175; doi:10.1038/s41467-019-11793-7)
Supplement: Supplementary file 2 — Reporting Summary [file 41467_2019_11793_MOESM2_ESM.pdf]

nature research

Corresponding author(s): Liming Liu
Last updated by author(s): Jul 29, 2019

Reporting Summary

Nature Research wishes to improve the reproducibility of the work that we publish. This form provides structure for consistency and transparency in reporting. For further information on Nature Research policies, see Authors & Referees and the Editorial Policy Checklist .

Statistics

For all statistical analyses, confirm that the following items are present in the figure legend, table legend, main text, or Methods section.

n/a	Confirmed	
☐	☒	The exact sample size (*n*) for each experimental group/condition, given as a discrete number and unit of measurement
☐	☒	A statement on whether measurements were taken from distinct samples or whether the same sample was measured repeatedly
☐	☒	The statistical test(s) used AND whether they are one- or two-sided<br>*Only common tests should be described solely by name; describe more complex techniques in the Methods section.*
☒	☐	A description of all covariates tested
☒	☐	A description of any assumptions or corrections, such as tests of normality and adjustment for multiple comparisons
☐	☒	A full description of the statistical parameters including central tendency (e.g. means) or other basic estimates (e.g. regression coefficient) AND variation (e.g. standard deviation) or associated estimates of uncertainty (e.g. confidence intervals)
☒	☐	For null hypothesis testing, the test statistic (e.g. *F*, *t*, *r*) with confidence intervals, effect sizes, degrees of freedom and *P* value noted<br>*Give P values as exact values whenever suitable.*
☒	☐	For Bayesian analysis, information on the choice of priors and Markov chain Monte Carlo settings
☒	☐	For hierarchical and complex designs, identification of the appropriate level for tests and full reporting of outcomes
☒	☐	Estimates of effect sizes (e.g. Cohen's *d*, Pearson's *r*), indicating how they were calculated

Our web collection on statistics for biologists contains articles on many of the points above.

Software and code

Policy information about availability of computer code

Data collection	Microsoft Excel, Agilent Chemstation, SoftMax pro
Data analysis	Origin 2019 64 bit, Microsoft Excel 2016, FlowJo VX

For manuscripts utilizing custom algorithms or software that are central to the research but not yet described in published literature, software must be made available to editors/reviewers. We strongly encourage code deposition in a community repository (e.g. GitHub). See the Nature Research guidelines for submitting code & software for further information.

Data

Policy information about availability of data

All manuscripts must include a data availability statement. This statement should provide the following information, where applicable:
- Accession codes, unique identifiers, or web links for publicly available datasets
- A list of figures that have associated raw data
- A description of any restrictions on data availability

Plasmid sequences needed in this study have been deposited in the databases of GenBank with corresponding accession number. Original data that supports the findings are available in the Source Data or from the corresponding authors upon reasonable request.

Field-specific reporting

Please select the one below that is the best fit for your research. If you are not sure, read the appropriate sections before making your selection.

☒ Life sciences ☐ Behavioural & social sciences ☐ Ecological, evolutionary & environmental sciences

Life sciences study design

All studies must disclose on these points even when the disclosure is negative.

Sample size	No sample size calculation was performed. As indicated in the text, all experiments were performed from a single colony, which provides some limited information about the distribution of the measurements and is typical of similar experiments and studies in the field. For flow cytometry, we analyzed 10000 cells / sample.
Data exclusions	We did not exclude any data.
Replication	All experimental findings are reliably reproducible. We have reproduced each experiment at least twice to ensure reliability, and have encountered no problems with reproducibility.
Randomization	Bacterial colonies from transformations were selected randomly. Allocation into experimental groups is irrelevant; single colonies were propagated as bacterial cultures, which were then split and tested with various experimental conditions, where applicable.
Blinding	Blinding is not relevant to our study because none of our data is based on qualitative scoring metrics nor does it involve animals or human research participants. As described in the above section for randomization, blinding during group allocation is irrelevant because the samples of bacterial cultures that were split into different conditions were random samplings and there is no control over which cells will be selected and thus, no bias during group allocation.

Reporting for specific materials, systems and methods

We require information from authors about some types of materials, experimental systems and methods used in many studies. Here, indicate whether each material, system or method listed is relevant to your study. If you are not sure if a list item applies to your research, read the appropriate section before selecting a response.

Materials & experimental systems

n/a	Involved in the study	
☒	☐	Antibodies
☒	☐	Eukaryotic cell lines
☒	☐	Palaeontology
☒	☐	Animals and other organisms
☒	☐	Human research participants
☒	☐	Clinical data

Methods

n/a	Involved in the study	
☒	☐	ChIP-seq
☐	☒	Flow cytometry
☒	☐	MRI-based neuroimaging

Flow Cytometry

Plots

Confirm that:

- ☒ The axis labels state the marker and fluorochrome used (e.g. CD4-FITC).
- ☒ The axis scales are clearly visible. Include numbers along axes only for bottom left plot of group (a 'group' is an analysis of identical markers).
- ☒ All plots are contour plots with outliers or pseudocolor plots.
- ☐ A numerical value for number of cells or percentage (with statistics) is provided.

Methodology

Sample preparation	Sample cells were washed twice with PBS and re-suspended to an OD600 of 0.2.
Instrument	LSRFortessaTM LSRII cell analyzer (BD Biosciences)
Software	FlowJo VX software (FlowJo, LLC)
Cell population abundance	at least 10,000 counts were recorded using a 0.5 mL s-1 flow rate.
Gating strategy	For flow cytometry analysis, cells were washed twice with PBS and re-suspended to an OD600 of 0.2. The assays were performed using a LSRFortessaTM LSRII cell analyzer (BD Biosciences) using FITC (GFP) and PE-TxRed (mCherry) channels. The voltage gains for each detector were set to: FITC, 407 V; PE-TxRed, 650 V. Compensation was performed using cells that express only GFP or mCherry. For each sample, at least 10,000 counts were recorded using a 0.5 mL s-1 flow rate. All data were exported in FCS3 format and processed using FlowJo software (FlowJo, LLC). A gate was previously designed based on forward and side scatter

graphs to remove debris from the analysis.

☒ Tick this box to confirm that a figure exemplifying the gating strategy is provided in the Supplementary Information.
