# Supplementary material for: Programmable biomolecular switches for rewiring flux in Escherichia coli
Source: Nat Commun. 2019 Aug 21;10:3751. doi: 10.1038/s41467-019-11793-7 (PMC6704175; doi:10.1038/s41467-019-11793-7)
Supplement: Supplementary file 3 — Description of Additional Supplementary Files [file 41467_2019_11793_MOESM3_ESM.docx]

**Description of Additional Supplementary Files**

**File Name:** Supplementary Data 1

**Description:** DNA sequences for plasmid genetic parts, proteases, reporter protein, and pathway enzymes.
